# MAP3K15 facilitates multiple viral genes expression in crustaceans via Dorsal-CC-CL-STAT axis besides the JNK/P38 pathway

**DOI:** 10.1371/journal.ppat.1013349

**Published:** 2025-08-01

**Authors:** Xiao-Qin Ran, Chen-Chen Liu, Xue-Mei Xu, Xin-Meng Wu, Cui-Jie Kang

**Affiliations:** Shandong Provincial Key Laboratory of Animal Cells and Developmental Biology, School of Life Sciences, Shandong University, Qingdao, Shandong, China; Xiamen University, CHINA

## Abstract

The mitogen-activated protein kinase (MAPK) pathway is a conserved signaling system that responds to extracellular signals and translates them into appropriate cellular responses. While multiple MAPK kinase kinases (MAP3Ks) play a crucial role in the step wise transmission of MAPK signals in response to the pathogen infection, little is known about the function of MAP3K15 (also known as apoptosis signal-regulated kinase 3, ASK3) in viral infection. Here, we provide evidence that shrimp MAP3K15 undergoes phosphorylation and activation during a DNA virus, white spot syndrome virus (WSSV) infection, the activated MAP3K15 interacted with the NF-кB homolog, Dorsal, to promote its nuclear translocation for expression of the coiled-coil-containing C-type lectin (CC-CL) and the viral immediate early (*ie*) genes. CC-CL then activates the JAK/STAT pathway as the ligand to its membrane receptor Domeless, driving the expression of more *ie* genes. In addition, the JNK/P38 signaling pathway is also activated to promote viral *ie* genes expression. Importantly, the viral amplification in a wide range of crustaceans were inhibited and the survival rates of host were improved effectively by suppressing MAP3K15 expression or utilizing SDK1, an inhibitor targeting the active form of MAP3K15, suggesting that the MAP3K15 has a critical and conserved function in viral infection. Taken together, this study elucidates a pivotal role and mechanism of MAP3K15 in DNA virus infection, providing novel insights and potential strategies for the control of WSSV infection in crustaceans aquaculture practice.

## Author summary

Innate immunity is the first line of defense against pathogens, and an important defense mechanism for vertebrates, especially lower vertebrates, to resist the invasion of external pathogens. The NF-κB, JAK/STAT, and MAPK signaling pathways play dual roles in the innate immune process of invertebrates, defending against pathogens while being susceptible to hijacking by pathogens to facilitate infection. However, the crosstalk and spatio-temporal dynamics of these pathways in response to pathogen invasion remain unclear. This study identifies MAP3K15 (also known as ASK3) as a key target of the White Spot Syndrome Virus (WSSV), acting as an initiator that links the NF-κB, JAK/STAT, and JNK/P38 pathways. MAP3K15 enhances the transcription of multiple viral immediate early (**ie **) genes, promoting WSSV replication. In addition, the compound SDK1, which inhibits MAP3K15 activation, effectively suppresses WSSV replication in several economically significant crustacean species. These findings suggest that the role of MAP3K15 in virus infection is conserved across different species and provides valuable insights into potential strategies for controlling viral diseases in crustacean aquaculture.

## Introduction

Animal life is constantly challenged by various threats, such as pathogen infections and environmental stressors. To maintain homeostasis, several evolutionarily conserved signal transduction pathways have developed to relay extracellular information into cellular responses. These pathways include the nuclear factor kappa-light-chain-enhancer of activated B cells (NF-κB) pathway, the Janus kinase-signal transducer and activator of transcription (JAK-STAT) pathway, and the mitogen-activated protein kinase (MAPK) cascade [1–3]. These pathways play crucial roles in the induction of inflammation, and the innate and adaptive immune responses to pathogens, making them primary targets for viral modulation [4].

During the early stages of viral infection, NF-κB and JAK/STAT signaling are often activated or manipulated to optimize viral replication. For instance, in the early phase of infection, the glycoprotein gp350 of Epstein-Barr virus (EBV) binds to cellular receptors CD21 and TLR2, resulting in the persistent activation of the classical NF-κB pathway [5]. Similarly, the oncoprotein E7 of high-risk human papillomavirus (HPV) suppresses STAT1 expression while activating STAT5 phosphorylation, promoting HPV replication via activation of the ATM DNA damage pathway [6,7]. Moreover, many viruses strategically incorporate NF-κB or STAT binding sites in their gene promoters, utilizing the activated pathways to promote viral gene expression, as seen in the human immune deficiency virus type 1 (HIV-1), human T-lymphotropic virus 1 (HTLV-1), hepatitis B virus (HBV), hepatitis C virus (HCV), rotaviruses, influenza viruses, and respiratory syncytial viruses (RSVs) [8–11]. Despite extensive research, the relationship of the two pathways on viral gene expression at various stages of infection remains inadequately understood, highlighting the need for further investigation into their interconnections.

WSSV, a highly pathogenic double-stranded DNA virus that infects a wide range of crustaceans, has the potential to cause up to 100% mortality in hosts within a week, leading to devastating economic losses in aquaculture [12,13]. During DNA virus invasion, the transcriptional activation of immediate early (*ie*)  genes by host immune-related transcription factors, such as NF-κB, STAT, or c-Jun, is a critical initial step [14–16].The activation of *wsv069* (*ie1*) expression has been attributed to the transcription factors of NF-κB-related signaling pathways (Toll/IMD), JNK/P38, or JAK/STAT separately in the publications, which contribute to WSSV amplification [17–19].The *ie1* gene product IE1 functions as a transcription factor with transactivation, dimerization, and DNA-binding activities, playing a pivotal role in regulating the expression of genes associated with DNA replication [20]. IE1 can also bind to the host JNK protein, enhancing its kinase activity toward the substrate c-Jun, thereby promoting the expression of* ie1* itself as well as other viral genes such as *wsv056*, *wsv249*, and *wsv403* [8]. This establishes a viral gene-mediated positive feedback loop that facilitates viral amplification. These findings indicate that the *ie1* gene is multi-functional and essential for viral invasion. In addition to *ie1*, the WSSV genome encodes over 20* ie *genes [21,22]. Dual-luciferase reporter assays in insect or mammalian cells have demonstrated that multiple ectopically expressed host proteins can upregulate the promoter activity of *ie *genes [23,24], however, the transcriptional regulation and functional roles of most IE genes within the host context remain largely unclear.

Viral invasion often triggers cellular stresses, including oxidative stress, ribosomal stress, and inflammatory cytokines, along with extracellular stimuli such as ultraviolet radiation [25]. These stimuli activate the MAPK pathway, a highly conserved signaling system across species that orchestrates optimal stress responses [26]. MAPK pathway regulates downstream transcription factors and controls gene expression through a three-tier kinase cascade involving MAPK kinase kinases (MAP3Ks), MAPK kinases (MAP2Ks), and MAPKs [27]. In humans, 24 different MAP3Ks (MAP3K1-21, A-Raf, Raf 1and B-Raf) have been identified, with significant diversity in their sequences except for conserved serine/threonine kinase domains critical for their regulatory activity [28]. The activation of MAP3Ks determines the specificity and function of MAPK pathways. Progressively increasing data show that some mammalian MAP3Ks participate in antiviral immunity through regulating type I interferon production [e.g., MAP3K2, MAP3K3, MAP3K5, MAP3K8, MAP3K11, MAP3K14, and Raf-1] and/or NF-κB signaling (e.g., MAP3K7 and MAP3K14) [29]. However, the roles of other MAP3Ks in antiviral immunity remain largely unexplored.

The apoptosis signal-regulating kinase (ASK) family, comprising ASK1, ASK2, and ASK3, belongs to the MAP3K family and is implicated in apoptotic cell death, stress responses, and various diseases [30,31]. Under resting conditions, ASK1 (MAP3K5) is maintained in an inactive state through interaction with inhibitory proteins such as reduced thioredoxin (TRX1) [32]. Upon oxidative stress or pathogen challenge, dissociation of these inhibitors permits ASK1 autophosphorylation, leading to the activation of MAP2Ks and subsequently the JNK and P38 MAPK pathways [33]. This cascade regulates proinflammatory cytokine production and apoptosis, thereby facilitating pathogen clearance and preserving tissue homeostasis [33]. ASK2 (MAP3K6), which shares high sequence homology with ASK1, often forms heteromeric complexes with ASK1 to amplify stress signals and enhance inflammatory and immune responses [34]. MAP3K15, primarily mediates osmotic stress responses, regulates apoptosis, and contributes to tissue homeostasis [35,36]. Although MAP3K15 is reported to be associated with phenotypic changes in bees and implicated in diseases such as diabetes and neurodegenerative disorders [37–39], the mechanisms for that are still largely unknown, as well as its role in the immune response.

This study elucidates the critical role and underlying mechanisms of MAP3K15 in the transcriptional regulation of *ie *genes within the WSSV genome. By interacting with Dorsal, MAP3K15 facilitates its nuclear translocation, inducing CC-CL expression and subsequently activating the JAK/STAT pathway to promote downstream viral gene expression. Through its ability to link the JNK/P38, NF-κB, and JAK/STAT pathways, MAP3K15 facilitates efficient WSSV replication in crustaceans. Furthermore, we evaluate the potential of SDK1, a compound that inhibits phosphorylation of MAP3K15, as a therapeutic strategy for managing WSSV infections in a wide range of crustaceans, disclosing its promise in antiviral interventions.

## Results

### MAP3K15 responds to and facilitates WSSV infection

**Fig 1:**
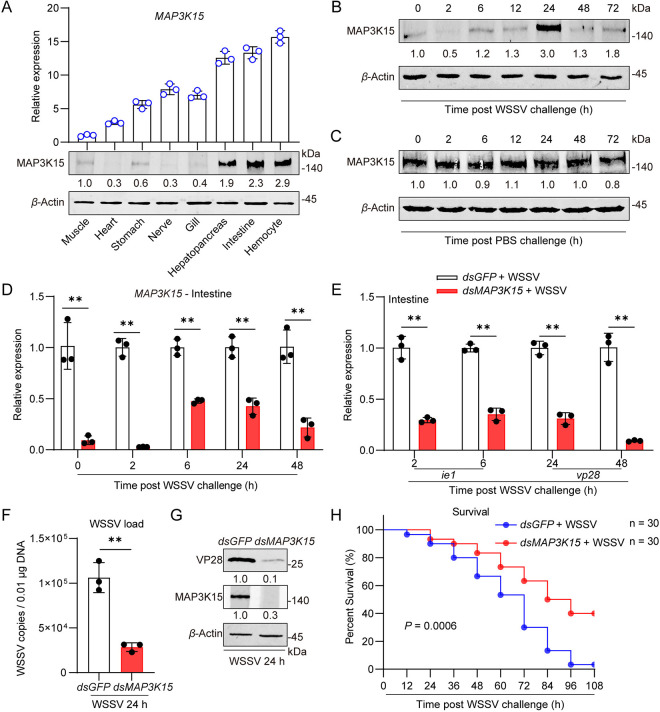
MAP3K15 facilitates WSSV infection. (A) Tissue distribution of MAP3K15 detected by qPCR (top) and western blot (bottom). (B, C) Western blot analysis of MAP3K15 protein levels in shrimp intestine at 0, 2, 6, 12, 24, 48, and 72 hours post-infection with WSSV (B) or PBS (C). (D) RNAi efficiency of MAP3K15 knockdown in the intestine assessed by qPCR. (E) The *ie1* and *vp28* expression levels at 2, 6, 24, and 48 (hpi) in the intestine of MAP3K15-knockdown and *dsGFP* control shrimp were analyzed by qPCR. (F) WSSV copy numbers in MAP3K15-knockdown and *dsGFP* shrimp were analyzed by qPCR using genomic DNA from the intestine. (G) The efficiency of MAP3K15-RNAi and VP28 protein levels in shrimp intestine was detected by western blot. (H) Survival rates of MAP3K15-knockdown shrimp following WSSV challenge (30 shrimp per group). The survival assay was analyzed using the Log-rank (Mantel-Cox) test. Data are presented as the mean ± SD from three independent replicates and were analyzed by Student’s *t*-test.**, *P *< 0.01. *β*-actin served as the internal reference for all the qPCR. Western blot bands were quantified using ImageJ. Relative expression levels of MAP3K15 or VP28/*β*-Actin were expressed as the numbers, and the value of the control group was set as one. *β*-Actin was used as the internal reference.

Based on previous transcriptome analyses, a gene with rapid response to WSSV infection and sustained up-regulated expression in the *Marsupenaeus japonicus* was chosen for in-depth study (S1A–S1C Fig), and named MAP3K15 (GenBank accession no. PQ660860) due to its homology to the MAP3K15 gene in mammals. Sequence alignment and phylogenetic analyses showed that MAP3K15 contains two conserved structural domains of the MAP3K family [Pfam: DUF4071 and Serine/Threonine protein kinases, catalytic domain (S-TKc)] and clusters with invertebrate MAP3K15 (S2 and S3 Figs). Since MAP3K15 sequence homology between shrimp and mammals is low, polyclonal antibodies to shrimp MAP3K15 were prepared (S1D and S1E Fig). Western blot and quantitative real-time polymerase chain reaction (q-PCR) analyses demonstrated that MAP3K15 was ubiquitously expressed across all tested tissues, with notably higher expression levels in the intestine, hepatopancreas, and hemocytes (Fig 1A). Western blot analysis revealed that MAP3K15 protein levels in the intestine progressively increased during WSSV infection, peaking at 24 h and subsequently declining, compared to the PBS control group (Fig 1B and 1C). Similarly, MAP3K15 levels in hemocytes also increased throughout WSSV infection, reaching a maximum at 24 h before gradually decreasing (S1F Fig).

Next, the *in vivo* function of MAP3K15 during WSSV infection was explored. RNA interference (RNAi) was performed to knock down MAP3K15 expression, and the RNAi efficiency was confirmed by detecting the expression of MAP3K15 in the intestine (Fig 1D) and hemocytes (S1G Fig), which were down-regulated more than half of that in the control group and continued to 48 hours post-infection. The expression of *ie 1* and the envelope protein (*vp*) 28 was decreased in the intestine (Fig 1E) and hemocytes (S1H Fig) of the MAP3K15-silenced group compared with the control group after WSSV infection. Moreover, shrimp in the MAP3K15-silenced group had lower viral loads (Fig 1F and 1G) and higher survival rates (Fig 1H) compared to shrimp in the control group, while no significant differences were observed between the two groups in the absence of viral challenge (S1I Fig). These data suggest that MAP3K15 promotes the amplification of WSSV in shrimp.

### MAP3K15 promotes the viral gene expression in a Dorsal-dependent manner

**Fig 2:**
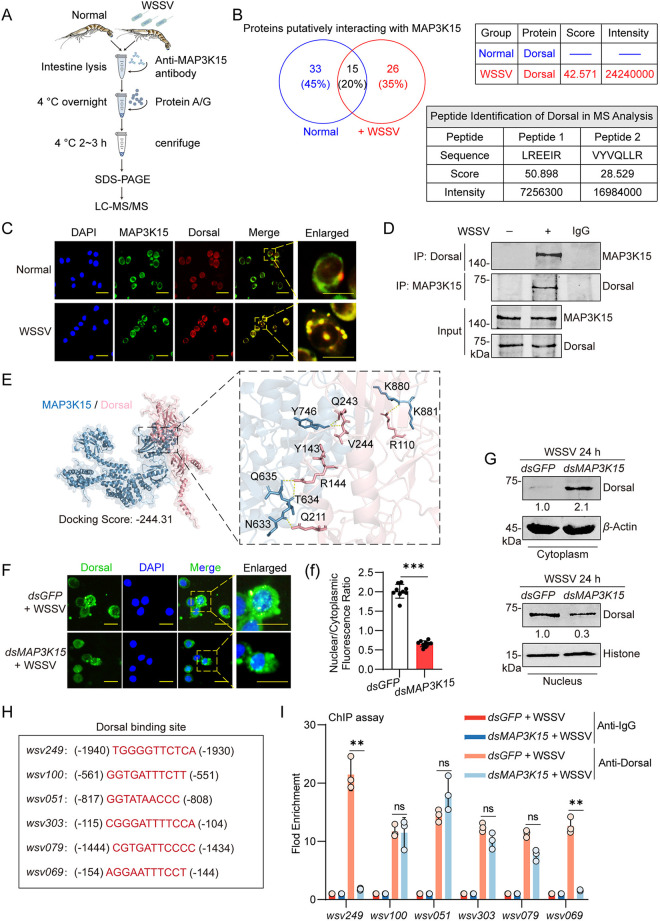
MAP3K15 interacts with the transcription factor Dorsal facilitates the viral *ie *genes expression. (A) Schematic diagram of experimental procedure for MAP3K15 immunoprecipitation and mass spectrometry. (B) The number of MAP3K15-interacting proteins identified by LC-MS/MS analysis is shown (left). Detailed information on Dorsal-related peptide identification is listed in the table (right). (C) Immunocytochemical analysis of MAP3K15 and Dorsal colocalization in uninfected and WSSV-infected shrimp hemocytes, with MAP3K15 labeled by FITC (green) and Dorsal by Fluor-647 (red). (D) Immunoprecipitation of MAP3K15 and Dorsal in shrimp intestine post-WSSV infection analyzed by western blot. (E) The HDOCK-predicted interaction model of MAP3K15 and Dorsal was visualized using PyMol. Structure-based protein interaction interface analysis between MAP3K15 and Dorsal. The cartoon represents the predicted MAP3K15-Dorsal complex structure, where the interaction hotspot residues are labeled. (F) The subcellular localization of Dorsal in the hemocytes of shrimp after 24 h post-WSSV challenge visualized by immunocytochemistry. Shrimp were pretreated with *dsGFP* or *dsMAP3K15*, respectively. Scale bars = 20 μm. (f) The nuclear-to-cytoplasmic fluorescence intensity ratio of Dorsal was quantitatively analyzed using ImageJ software. (G) Dorsal distribution cytoplasmic and nuclear fractions of intestine was assessed by western blot. *β*-Actin and Histone served as internal controls. (H) Predicted Dorsal binding sites on* ie* gene promoters analyzed using Promoter 3.0 and JASPAR. (I) The binding of Dorsal to the *ie* gene promoter regions in hemocytes at 6 h post-WSSV infection was detected by qPCR after ChIP. Shrimp were pretreated with *dsGFP* or *dsMAP3K15*, respectively. Data are presented as the mean ± SD from three independent replicates and were analyzed by Student’s *t*-test. ns, no significant difference, **, *P *< 0.01. *β*-actin was used as the internal reference for all the qPCR. Western blot bands were quantified using ImageJ; the resulting values represent relative band intensities normalized to the control group (set to 1).

To understand the mechanism of shrimp MAP3K15 during WSSV proliferation, we performed co-immunoprecipitation (Co-IP) analysis plus liquid chromatography-tandem mass spectrometry (LC-MS/MS) analysis to identify changes in proteins that interact with MAP3K15 before and after WSSV infection (Figs 2A and S4A). In the normal group and the WSSV-infected group, 33 proteins and 26 proteins were identified, respectively. Among them, Dorsal, a transcription factor of the NF-кB signaling pathway in shrimp, was identified as a MAP3K15-interacting protein involved in the WSSV infection (Fig 2B). Immunofluorescence co-localization and *in vivo* Co-IP assays revealed that the interaction between MAP3K15 and Dorsal was detected only in the WSSV-infected group, but not in the normal controls, suggesting that this interaction is induced by viral infection (Fig 2C and 2D). Analysis using the HDOCK and PyMol tool found that MAP3K15 (1–1130 aa) and Dorsal (1–686 aa) form a protein-protein interaction interface with a docking score of -244.31 (Fig 2E), indicating that they are interaction partners. Subsequently, whether MAP3K15 was needed for the nucleus translocation of Dorsal during WSSV infection was tested by RNAi. Immunocytochemistry results showed that silencing MAP3K15 significantly suppressed WSSV-induced nuclear accumulation of Dorsal compared to the control group (Figs 2F(f) and S4B(b)). Consistently, western blot analysis also demonstrated that knockdown of MAP3K15 reduced the nuclear expression of Dorsal upon WSSV infection, suggesting that the interaction between MAP3K15 and Dorsal may facilitate the nuclear translocation of Dorsal.

Since the transcription of viral *ie* genes is usually the first step in viral infection and is activated by exploiting the host NF-кB signaling pathway [40], we supposed that the nuclear translocation of Dorsal promotes transcription of the viral *ie *genes. By searching the published literature, all the reported *ie *genes of WSSV were used for Dorsal binding site searching via JASPAR software [22,41], then the promoter sequence of six candidates *ie* genes (*wsv051*, *wsv249*, *wsv069*, *wsv079*, *wsv100* and *wsv303*) was cloned and chromatin immunoprecipitation (ChIP) assay was performed to test their binding activity to Dorsal *in vivo*. As shown in Fig 2H and 2I, Dorsal could interact with DNA fragments containing the proposed Dorsal-binding site in all the tested viral* ie *genes. Notably, the interaction of Dorsal with *wsv249* or *wsv069* was eliminated upon knockdown of MAP3K15 in the Anti-Dorsal group, whereas the other genes remained unchanged, suggesting that the transcription of these two *ie*  genes was MAP3K15-dependent. Taken together, these data suggested that the MAP3K15 is involved in the regulation of viral *ie *genes expression through binding to and promoting the nuclear translocation of Dorsal, a transcription factor of the host NF-кB signaling pathway.

### MAP3K15 regulates the nuclear translocation of STAT and the expression of viral *ie * genes

**Fig 3:**
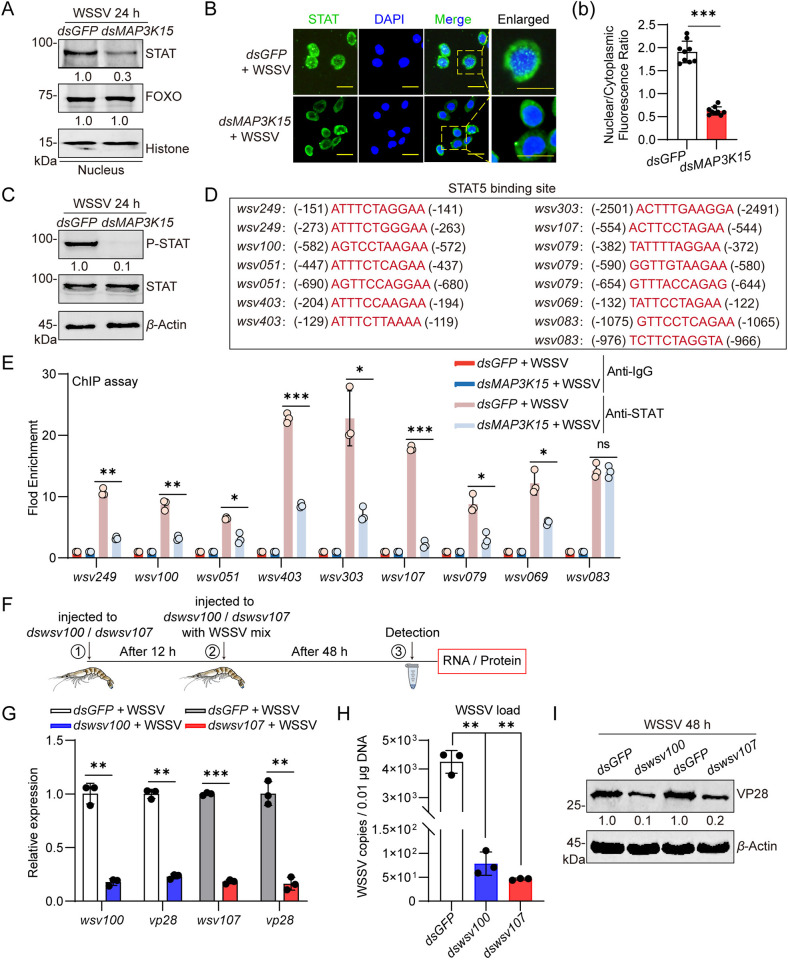
Identified the viral *ie* genes regulated by STAT and their function *in vivo.* (A) The nuclear distribution of STAT and FOXO in the intestine at 24 h post-WSSV challenge was detected by western blot. (B) Nuclear translocation of STAT in hemocytes at 24 h post-WSSV infection analyzed by immunocytochemistry. Scale bars = 20 μm. (b) The nuclear-to-cytoplasmic fluorescence intensity ratio of STAT was quantitatively analyzed using ImageJ software. (C) STAT phosphorylation in the intestine was detected by western blot. Shrimp were pretreated with *dsGFP* or *dsMAP3K15*. (D) Predicted STAT binding sites on *ie* gene promoters. (E) The binding of STAT to the *ie*  gene promoter regions in hemocytes at 6 h post-WSSV challenge was detected by qPCR after ChIP assay. Shrimp were pretreated with *dsGFP* or *dsMAP3K15*. (F) Schematic diagram of experimental procedure. (G) The expression efficiency of *wsv100*-RNAi and *wsv107*-RNAi in the intestine and the expression level of *vp28* at 48 h post-WSSV challenge were detected by qPCR. (H) WSSV copy numbers in *wsv100*, *wsv107*-knockdown, and *dsGFP* shrimp were analyzed by qPCR using genomic DNA from the intestine. (I) The expression level of VP28 protein after the knockdown of *wsv100* or *wsv107* in shrimp intestine was determined by western blot. Data are presented as the mean ± SD from three independent replicates and were analyzed by Student’s *t*-test. ns, no significant difference, *, *P* < 0.05, **, *P *< 0.01. ***, *P *< 0.001. *β*-actin served as the internal reference for all the qPCR. Western blot bands were quantified using ImageJ. The numbers indicate the relative band intensities. *β*-Actin, Histone was used as an internal reference.

In addition to the NF-кB pathway, the JAK-STAT and PP2A-FOXO pathways have also been reported to function in activating the expression of viral *ie*  genes (*ie* *1* and *wsv079* separately) in shrimp [19,42]. Therefore, we examined the nuclear distribution of STAT, and FOXO before and after MAP3K15 knockdown to understand whether MAP3K15 regulates the viral *ie *genes expression through those pathways. Western blot results showed that under WSSV infection, nuclear localization of STAT was markedly reduced in the *dsMAP3K15* group compared to the *dsGFP* group, while FOXO expression remained unchanged between the two groups (Fig 3A). Under normal conditions, injection of either *dsMAP3K15* or *dsGFP* had no effect on the nuclear distribution of STAT or FOXO (S5A Fig). The same results occurred in the immunocytochemistry assay (Figs 3B (b) and S5B (b)). Moreover, the viral infection-induced STAT phosphorylation was abolished in the *dsMAP3K15* group but not in the control group (Fig 3C), it was not changed under the normal conditions (S5C Fig). These results suggested that the MAP3K15 participates in the JAK-STAT pathway but not the PP2A-FOXO pathway. Thus, the ChIP assay combined MAP3K15 silencing was performed to test whether the binding of STAT to more *ie* genes occurs and which one is regulated by MAP3K15 *in vivo*. The results showed that the binding of STAT to *ie *genes (*wsv249*, *wsv100*, *wsv051, wsv403, wsv304, wsv107, wsv069, wsv083*) existed during viral infection and all of them were inhibited by MAP3K15 knockdown except *wsv083* (Fig 3D and 3E).

To confirm whether the *ie* genes downstream of MAP3K15 are critical for viral infection in shrimp, four* ie*  genes (*wsv100*, *wsv107*, *wsv069*, and *wsv249*) were selected for functional analysis based on the results shown in Figs 2I and 3E. RNAi experiments were conducted as illustrated in Fig 3F. The results showed that both the expression of VP28 and the viral titer decreased obviously in the *wsv100* and *wsv107* silencing group compared with the control (Fig 3G–3I), indicating they are necessary for viral infection in shrimp. Similar results were observed in the *wsv069* and *wsv249* knockdown assays (S5D–S5G Fig).

### MAP3K15/Dorsal bridges the NF-кB and JAK/STAT signaling pathways via regulates CC-CL expression

**Fig 4:**
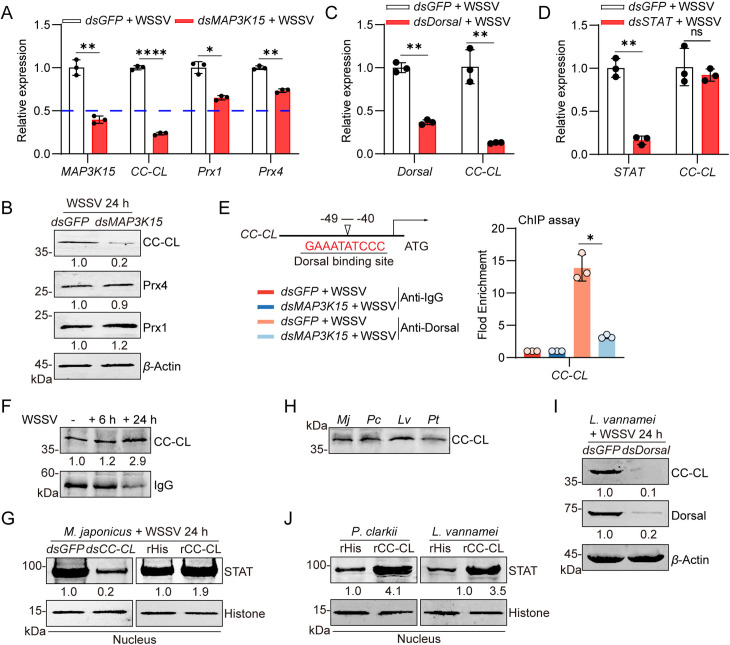
MAP3K15/Dorsal induces CC-CL expression and activates the JAK/STAT pathway in decapods. (A, B) The expression levels of CC-CL, Prx1, and Prx4 in the intestine following MAP3K15 knockdown under WSSV infection were detected by qPCR (A) and western blot (B). (C, D) qPCR analysis of CC-CL expression in shrimp intestine following knockdown of Dorsal (C) or STAT (D) under WSSV infection. (E) Schematic diagram of the predicted binding sites of Dorsal on the *CC-CL* promoter. The binding of Dorsal to the *CC-CL* promoter region in hemocytes at 6 h post-WSSV challenge was detected by qPCR after ChIP. Shrimp were pretreated with *dsGFP* or *dsMAP3K15*. (F) Western blot analysis of CC-CL protein levels in shrimp hemolymph under normal conditions and at various time points post-WSSV infection. (G) Western blot analysis of nuclear STAT levels in the intestine of *M. japonicus* following WSSV infection under CC-CL knockdown or overexpression. (H) Western blot detection of CC-CL expression in *M. japonicus*, *L. vannamei*, *P. clarkii*, and *P. trituberculatus*. (I) Western blot analysis of CC-CL expression in the intestine of *L. vannamei* following Dorsal knockdown and WSSV infection. (J) Western blot analysis of nuclear STAT distribution in the intestine of *L. vannamei* and *P. clarkii* after CC-CL overexpression. Data are presented as the mean ± SD from three independent replicates and were analyzed by Student’s *t*-test. ns, no significant difference, *, *P* < 0.05, **, *P *< 0.01. ****, *P *< 0.0001. *β*-actin served as the internal reference for all the qPCR. Western blot bands were quantified using ImageJ, and the relative intensities, normalized to the internal control, are presented as numerical values.

To explore the mechanism by which the MAP3K15 regulates the JAK/STAT pathway, we first examined whether MAP3K15 acts by affecting the expression of the ligands for the membrane receptor, Domeless, of the shrimp JAK/STAT pathway. In our previous research, peroxiredoxin 4 (Prx4) and the coiled-coil containing C-type lectin (CC-CL) have been proven to be the ligand for Domeless (the membrane receptor of JAK/STAT pathway in shrimp) and functioned in activating the JAK/STAT pathway for bacterial clearance in shrimp *M. japonicus* [43,44]. So, we first detected whether the expression of Prx4, Prx1(another Prx subfamily member, was used as control), and CC-CL was inhibited by MAP3K15 knockdown and response to WSSV infection. As shown in Fig 4A and 4B, silencing MAP3K15 led to a significant reduction in CC-CL expression at both the transcriptional and translational levels. In contrast, Prx1 and Prx4 showed only slight decreases in mRNA levels and no significant changes in protein expression. These results suggest that MAP3K15 primarily influences the JAK/STAT pathway by regulating CC-CL expression. Then, the expression pattern of CC-CL was detected and showed kept increasing during the viral infection (S6A Fig). The transcription of CC-CL was suppressed by Dorsal knockdown but not by STAT knockdown as shown in Fig 4C and 4D, suggesting that its expression was Dorsal dependent. Furthermore, the Dorsal binding site was found by searching the promoter sequence of CC-CL, and the binding of Dorsal with the DNA fragments of CC-CL was confirmed to be related to MAP3K15 through ChIP assay and MAP3K15 silencing (Fig 4E). Together, these findings demonstrate that the expression of CC-CL was coordinately regulated by the MAP3K15–Dorsal axis.

Next, we investigated whether CC CL could be secreted into the extracellular and act as a cytokine. Immunoprecipitation and Western blot analyses revealed that CC-CL protein was present in the hemolymph of shrimp under normal conditions, and its expression was up-regulated following WSSV infection (Fig 4F). To confirm whether CC-CL functions to activate the JAK/STAT pathway during WSSV infection, CC-CL was silenced or overexpressed (purified recombinant CC-CL protein, rCC-CL, was injected into shrimp to generate an overexpression-like effect) (S6B and S6C Fig). The STAT protein level in the nucleus was detected by western blot under two conditions. The results showed that the nuclear STAT protein was significantly inhibited by silencing CC-CL and enhanced by overexpression of CC-CL (Fig 4G), suggesting that CC-CL could effectively activate the JAK/STAT pathway. Collectively, these results suggest that MAP3K15/Dorsal-induced CC-CL expression during WSSV infection functionally links the NF-κB and JAK/STAT signaling pathways.

To determine whether the MAP3K15/Dorsal–CC-CL–STAT axis is conserved in other crustaceans, we first examined CC-CL expression in various decapod species. Western blot analysis using a polyclonal CC-CL antibody detected the presence of CC-CL protein in the hemolymph of *Litopenaeus vannamei*, *Procambarus clarkii*, and *Portunus trituberculatus* (Fig 4H). In *L. vannamei*, Dorsal knockdown significantly reduced CC-CL protein levels (Fig 4I). Furthermore, overexpression of CC-CL led to increased nuclear STAT protein levels in both *P. clarkii* and *L. vannamei* (Fig 4J). Collectively, these findings suggest that the MAP3K15/Dorsal–CC-CL–STAT axis is highly conserved among decapod.

### CC-CL facilitates WSSV infection through promoting the expression of STAT-dependent* ie* genes

**Fig 5:**
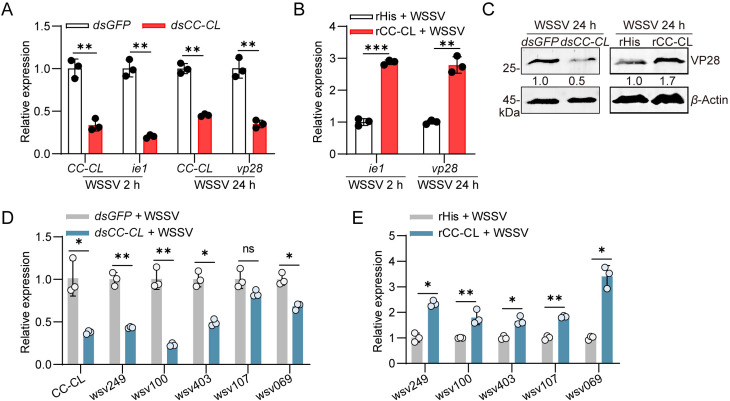
CC-CL promotes the STAT-dependent *ie* genes transcription. (A, B) The expression of *ie* *1* and *vp28* in shrimp intestine following *CC-CL* knockdown (A) or overexpression (B) during WSSV infection were detected by qPCR. *dsGFP* or rHis was used as control. (C) VP28 protein levels in shrimp intestine following *dsCC-CL* or rCC-CL injection were analyzed by western blot. (D, E) The expression of *wsv249*, *wsv100*, *wsv403*, *wsv107*, and *wsv069* in the intestine after 6 h post-WSSV infection was analyzed by qPCR. Shrimp were pretreated with *dsCC-CL* (D) or rCC-CL (E), respectively. *dsGFP* or rHis was used as control. Data are presented as the mean ± SD from three independent replicates and were analyzed by Student’s *t* - test. ns, no significant difference, *, *P* < 0.05, **, *P *< 0.01. ***, *P *< 0.001. *β*-actin served as the internal reference for all the qPCR. Western blot bands were quantified using ImageJ, with values representing relative band intensities (VP28/*β*-Actin), normalized to the control group.

To explore the function of CC-CL in WSSV infection, we determined WSSV proliferation in WSSV-infected shrimp after knockdown or overexpression of CC-CL. Compared with the control group (*dsGFP* treatment), the expression of the *ie1* and *vp28* genes significantly decreased after CC-CL silencing (Fig 5A). Overexpression of rCC-CL significantly increased the transcription of viral *ie* *1* and *vp28* compared with the control group (rHis supplement) (Fig 5B). Western blot analysis confirmed the same trend in the expression of VP28 protein levels (Fig 5C). This data suggested that the expression of CC-CL was beneficial for WSSV infection.

As the nuclear translocation of STAT was enhanced by CC-CL expression, we supposed that CC-CL functioned by facilitating the STAT-dependent* ie* genes’ expression. Thus, qPCR analysis was performed to test five selected *ie* genes (*wsv249*, *wsv100*, *wsv403*, *wsv107*, and *wsv069*) expression upon CC-CL silencing or overexpression in the WSSV-infection shrimp. Compared with the control group (*dsGFP* group), the expression of all the tested genes was down-regulated by CC-CL silencing, except the *wsv107* was unchanged (Fig 5D). In contrast, CC-CL overexpression markedly increased the transcript levels of all examined genes (Fig 5E). These results suggest that CC-CL promotes the expression of STAT-dependent *ie* genes to facilitate WSSV infection.

### SDK1 inhibits MAP3K15 phosphorylation and effectively suppresses WSSV replication in crustaceans

**Fig 6:**
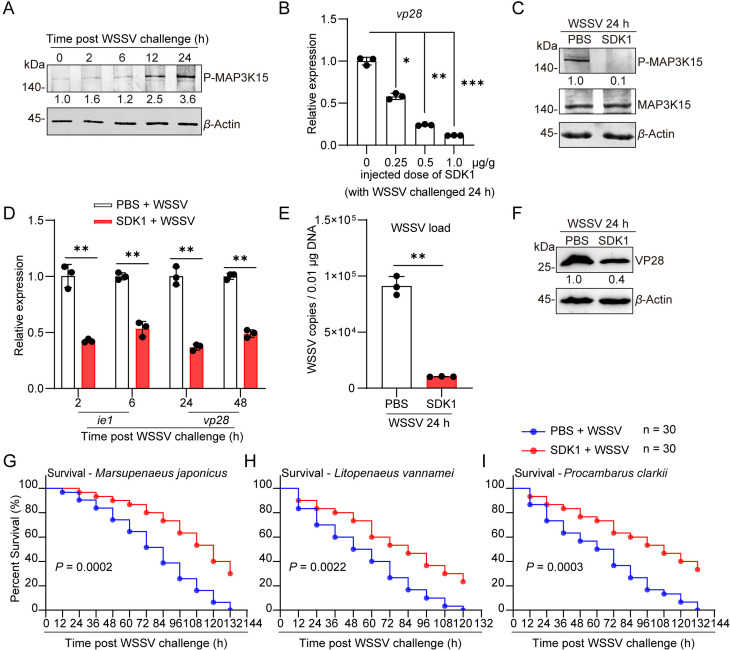
SDK1 inhibits MAP3K15 activity and WSSV infection in shrimp. (A) Phosphorylation levels of MAP3K15 in shrimp intestine at 2, 6, 12, and 24 hpi were analyzed by western blot. (B) The effect of different doses of SDK1 on WSSV replication was analyzed using *vp28* expression as an indicator. (C) MAP3K15 phosphorylation in the intestine was detected by western blot. Shrimp were pretreated with SDK1(0.5 g/g) or PBS for 2 h. (D) Expression levels of *ie1* and *vp28* at different time points post-WSSV infection in shrimp intestine of SDK1- or PBS-treated analyzed by qPCR. (E) The copy number of WSSV after SDK1 or PBS solvent treatment was detected by qPCR. (F) VP28 protein levels in the intestine after SDK1 or PBS treatment were detected by western blot. (G-I) The survival rate in *M. japonicus* (G), *L. vannamei* (H), and *P. clarkii* (I) after SDK1 application with WSSV challenge. PBS solvent injection was used as the control. The survival assay was analyzed using the Log-rank (Mantel-Cox) test, thirty shrimp were used in each group. Data are presented as the mean ± SD from three independent replicates and were analyzed by Student’s *t*-test. *, *P* < 0.05, **, *P *< 0.01. ***, *P *< 0.001. *β*-actin served as the internal reference for all the qPCR. Western blot bands were digitized using ImageJ. The numbers indicate the relative band intensities. *β*-Actin was used as an internal reference.

Phosphorylation of MAP3K15 in the shrimp intestine increased significantly with the duration of WSSV infection (Fig 6A), indicating that WSSV infection activates phosphorylation of MAP3K15 protein. To determine whether MAP3K15 phosphorylation is a determinant for WSSV expansion in shrimp hosts, one chemical compound (SDK1) was developed to inhibit MAP3K15 phosphorylation. The inhibitory effect of different concentrations of the SDK1 in shrimp showed that virus replication was significantly reduced with the increase of the concentration used, indicating that the compound could effectively reduce the replication of WSSV in shrimp (Fig 6B). We then chose the concentration with moderate virulence inhibitory activity (0.5 μg/g) for the latter experiments. At this concentration, the phosphorylation of MAP3K15 protein was effectively inhibited (Fig 6C). In *M. japonicus*, SDK1 treatment markedly reduced the expression of *ie1* and *vp28* in intestine and hemocytes compared with controls (Figs 6D and S7A), and significantly decreased WSSV viral titer and VP28 protein level (Fig 6E and 6F). It’s worth noting that, the transcription of *ie* *1* and *vp*28 genes in the other species, such as *L*. *vannamei*, *P*. *clarkii*, and *P*. *trituberculatus* were also inhibited when SDK1 was used (S7B and S7C Fig), indicating that it has a broad-spectrum effect of inhibiting viral replication. Subsequently, the *in vivo* function of SDK1 in three shrimp species including *M. japonicus*, *L. vannamei*, and *P. clarkii* was tested by mortality rate assay. Preliminary toxicity tests showed that SDK1 and PBS solvent treatments had no significant effect on the survival of the tested species under normal conditions (S7D–S7F Fig). However, SDK1 treatment significantly improved the survival of WSSV-infected shrimp compared to controls (Fig 6G–6I). The results showed that SDK1 treatment can significantly improve the survival rate of all the test species.

To understand whether SDK1 functions under immersing conditions, SDK1(at a final concentration of 40 nM) or mock solution was added into seawater with WSSV, and three hundred postlarvae (approximately 0.5 cm) were divided into three groups and used for survival assay. First, compared with the control group, the expression of *ie* *1* and *vp28* in the SDK1-treated group was significantly reduced as detected by qPCR (S7G Fig). In addition, the survival rate of postlarvae in the SDK1 treatment group was higher than that in the control group (S7H and S7I Fig). These data suggest that SDK1 also functioned in blocking the WSSV infection in the immersed condition. Taken together, the results confirmed that the WSSV-induced MAP3K15 phosphorylation is an important event for the virus amplification in hosts and SDK1 can effectively inhibit WSSV replication in a wide range of crustaceans.

### SDK1 inhibits the WSSV amplification by blocking the signal transduction downstream of MAP3K15

**Fig 7:**
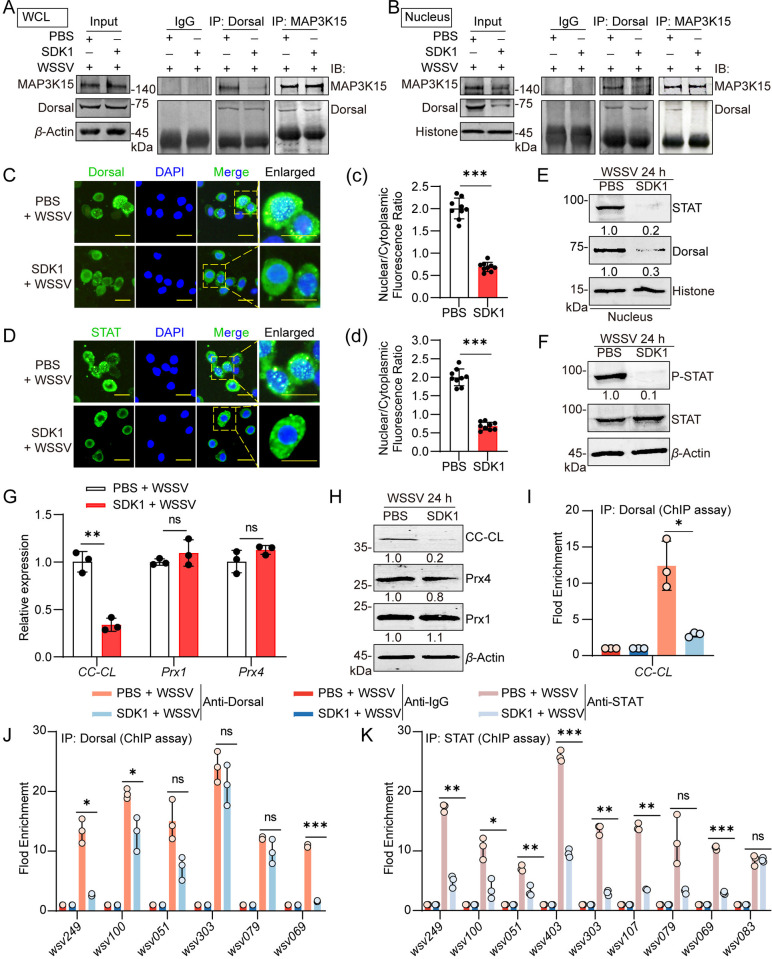
SDK1 inhibits MAP3K15/Dorsal, STAT nuclear translocation, inhibits CC-CL, and the *ie *genes expression. (A, B) Co‐immunoprecipitation and western blot analysis of MAP3K15–Dorsal interaction in total protein (A) and nuclear (B) extracts from shrimp at 24 h post‐WSSV challenge. (C, D) The subcellular localization of Dorsal (C) and STAT (D) in hemocytes at 24 h post-WSSV challenge visualized by immunocytochemistry. Shrimp were pretreated with PBS or SDK1, respectively. Scale bars = 20 μm. (c-d) The nuclear-to-cytoplasmic fluorescence intensity ratio of Dorsal (c) and STAT (d) was quantitatively analyzed using ImageJ software. (E-F) After SDK1 or PBS treatment, the distribution of Dorsal and STAT (E), and STAT phosphorylation in shrimp intestine (F) was detected by western blot. (G) The expression of *CC-CL*, *Prx1*, and *Prx4* in the intestine after SDK1 or PBS treatment and WSSV infection was analyzed by qPCR. (H) Western blot analysis of CC-CL, Prx1, and Prx4 expression in shrimp intestine following SDK1 or PBS treatment and WSSV infection. (I) The binding of Dorsal to the *CC-CL* promoter region in hemocytes after 6 h post-WSSV challenge was detected by qPCR after ChIP. Shrimp were pretreated with SDK1 or PBS. (J, K) The binding of Dorsal (J), and STAT (K) to the* ie* gene promoter regions was detected in hemocytes by qPCR after ChIP. Data are presented as the mean ± SD from three independent replicates and were analyzed by Student’s *t*-test. ns, no significant difference, *, *P* < 0.05, **, *P *< 0.01. ***, *P *< 0.001. *β*-actin served as the internal reference for all the qPCR. Western blot bands were quantified using ImageJ. The numbers indicate the relative band intensities. *β*-Actin, Histone was used as an internal reference.

To reveal the molecular mechanism of WSSV inhibition by SDK1, we first tested whether the phosphorylation of MAP3K15 was required for MAP3K15 interaction with Dorsal. The results showed that SDK1 treatment attenuated the virus infection-induced interaction of MAP3K15 with Dorsal when total protein was used for IP assays (Fig 7A). Interestingly when IP assays were performed using the cytoplasmic or nuclear proteins from intestinal tissues respectively, the interaction between MAP3K15 and Dorsal was more pronounced in the cytoplasm but attenuated in the nucleus in the SDK1-group compared with the control group (Figs 7B and S8A). These data showed that SDK1 inhibited the binding of phosphorylated MAP3K15 with Dorsal in the nucleus rather than in the cytoplasm. Immunocytochemistry and Western blot analyses demonstrated that SDK1 treatment significantly inhibited WSSV‑induced nuclear translocation of Dorsal and STAT and attenuated STAT phosphorylation levels (Fig 7C–7F). Consistent results were observed in *L. vannamei*, where SDK1 treatment reduced nuclear localization of Dorsal and STAT and suppressed STAT phosphorylation (S8B and S8C Fig). In addition, qPCR and Western blot analyses revealed that SDK1 treatment significantly suppressed both the transcription and protein expression of CC-CL, while the expression levels of Prx1 and Prx4 remained unchanged in *M. japonicus* (Fig 7G and 7H). Consistently, SDK1 treatment reduced CC‑CL protein levels in *L. vannamei* (S8D Fig). ChIP analysis further demonstrated that SDK1 treatment significantly impaired Dorsal binding to the CC‑CL promoter region (Fig 7I). Moreover, the binding of the Dorsal or STAT to the *ie* genes was down-regulated in the SDK1-treated group compared to the control group (Fig 7J and 7K). Similarly, SDK1 treatment significantly down-regulated the transcriptional levels of *wsv249*, *wsv100*, *wsv403*, *wsv107*, and *wsv069* in *L. vannamei* (S8E Fig). These data suggested that SDK1 inhibited the WSSV amplification via inhibiting the interaction of phosphorylated-MAP3K15 with Dorsal in the nucleus and blocking the subsequent Dorsal-CC-CL-STAT- *ie* axis signal transduction.

### MAP3K15 activates the JNK/P38 pathway for WSSV proliferation

**Fig 8:**
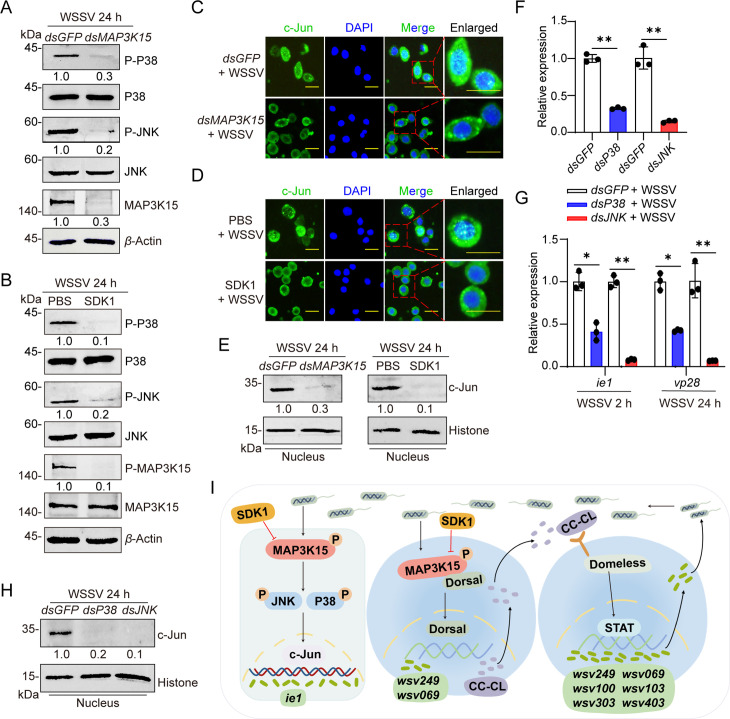
MAP3K15 promotes WSSV infection through the activated JNK/P38-c-Jun/IE pathway in shrimp. (A, B) The phosphorylation and total protein levels of MAP3K15, JNK, and P38 in shrimp intestine following *dsMAP3K15* (A) or SDK1 (B) injection were analyzed by western blot. *dsGFP* or PBS treatment was used as a control. (C, D) The subcellular localization of c-Jun in shrimp hemocytes following MAP3K15 knockdown (C) or SDK1 injection (D) after 24 h post-WSSV challenge was visualized by immunocytochemistry. Scale bars = 20 μm. (E) The expression of c-Jun in the nucleus of the shrimp intestine was detected by western blot after *dsMAP3K15* or SDK1 treatment. *dsGFP* or PBS treatment was used as a control. (F, G) RNAi efficiency (F) and the transcription of *ie1* and *vp28* (G) in shrimp intestine following JNK or P38 knockdown during WSSV infection was detected by qPCR. *dsGFP* injection was used as a control. (H) The expression of c-Jun in the nucleus of shrimp intestine after knockdown JNK/P38 was detected by western blot. (I) Mechanistic model of the study. WSSV activates MAP3K15, which interacts with Dorsal to promote Dorsal nuclear translocation and CC-CL expression. CC-CL activates the JAK/STAT signaling pathway, enhancing viral *ie* genes transcription. Additionally, MAP3K15 activates the JNK/P38 pathway, facilitating WSSV replication. Inhibitor SDK1 inhibits MAP3K15 activation and downstream signal, thereby inhibiting the WSSV replication in the host. Data are presented as the mean ± SD from three independent replicate and were analyzed by Student’s *t*-test. *, *P* < 0.05, **, *P *< 0.01. *β*-actin served as the internal reference for all qPCR analyses. Western blot bands were quantified using ImageJ. The numbers indicate the relative band intensities. *β*-Actin, Histone was used as an internal reference.

Due to the JNK and P38 mediate signal transduction are the classical downstream pathway of MAP3Ks, and the IE1/JNK/c-Jun positive feedback loop, as well as P38, has been reported to be involved in the amplification of WSSV in *L. vannamei* [18,45,46], we then detected whether MAP3K15 functions in activating those two pathways in *M. japonicus*. The results showed that the WSSV infection-induced phosphorylation levels of JNK or P38 were significantly decreased in the *MAP3K15* silencing group or SDK1-treated group compared to the control group (Fig 8A and 8B). Immunocytochemistry experiments showed that the knockdown of MAP3K15 or application of SDK1 inhibited the enrichment of c-Jun in the nucleus induced by WSSV infection (Fig 8C and 8D). Western blot analysis confirmed the results (Fig 8E). In addition, the expression of* ie1* and *vp28* was significantly reduced after the successful knockdown of JNK or P38 (Fig 8F and 8G), and the expression of the c-Jun protein in the nucleus was significantly decreased in the JNK or P38 silencing group compared with the control group (Fig 8H). These data suggested that the classical MAP3K15-JNK/P38-c-Jun- *ie*1 pathway was conserved in shrimp *M. japonicus,* which was also activated by WSSV infection and contributed to the viral infection.

## Discussion

This study elucidates the molecular mechanism by which MAP3K15 promotes viral infection. During WSSV infection, host MAP3K15 is activated and systematically manipulates the JNK/P38, NF-κB, and JAK/STAT immune-related signaling pathways, enabling the amplification of multiple viral genes and ultimately promoting viral replication. Targeting MAP3K15 by knockdown the gene expression or inhibition the activation of its protein contributes to the inhibition of viral amplification and improvement of survival rates in several crustaceans. These processes are illustrated in Fig 8I.

In mammals, MAP3K15 (ASK3) is known to participate in cell death, cell volume control, and disease development, with no reports on immune-related function while other ASK family members, such as ASK1 and ASK2, are well-established participants in cellular immune defense [35,47]. Functional investigation of the MAPKKK15 gene from *L. vannamei* revealed that *Lv*MAPKKK15 facilitates viral amplification by suppressing the promoter activities of several antiviral genes [48]. In addition, dual-luciferase reporter assays demonstrated that *Lv*MAPKKK15 could enhance the promoter activities of several *ie* genes in insect S2 cells. Our study supports and extends these findings by further elucidating how MAP3K15 regulates the transcription of specific *ie* genes (Fig 8I). Moreover, we showed that MAP3K15 promotes dsDNA virus (WSSV) replication across multiple crustacean species, including *M. japonicus*, *L. vannamei*, *P. clarkii*, and *P. trituberculatus*. Inhibition of MAP3K15 expression, either by RNAi or pharmacological treatment, significantly suppressed viral replication *in vivo* and improved host survival (Figs 6 and S7). This antiviral effect was mediated through both the Dorsal–CC–CL–STAT axis and the JNK/P38 pathway, indicating that the function of MAP3K15 is conserved in crustaceans, at least within the order decapoda. Moreover, shrimp MAP3K15 contains two conserved structural domains of the MAP3K family [Pfam: DUF4071 and Serine/Threonine protein kinases, catalytic domain (S-TKc)] and clusters with invertebrate MAP3K15 (S2 and S3 Figs). These findings suggest that the function and mechanism of MAP3K15 in antiviral responses may be conserved across invertebrate species, and further studies are needed to verify this.

The expression of immediate early proteins are critical regulators that manipulate host systems for viral replication [15,49]. Our findings demonstrate that Dorsal and STAT are essential for the transcription of most *ie* genes in shrimp. However, not all Dorsal or STAT-dependent *ie* genes transcription is regulated by MAP3K15, as shown in Figs 2I and 3E. For example, the expression of certain *ie* genes (*wsv051*, *wsv*079, *wsv*100, and *wsv*303) by Dorsal appears to be independent of MAP3K15, suggesting the involvement of alternative upstream signals in the Dorsal–*ie* axis (Fig 2I). In contrast, the transcription of most STAT-dependent *ie* genes was regulated by MAP3K15 (Fig 3E). Given that CC-CL expression depends on MAP3K15 activation and its interaction with Dorsal during WSSV infection (Figs 4D–4E and 7A–7B), and that CC-CL contributes to STAT nuclear translocation in multiple species (Fig 4G and 4J), we propose that the MAP3K15/Dorsal–CC–CL–STAT axis is a key pathway regulating *ie* genes expression in decapoda. In our research, the expression of *wsv069* and *wsv249* is jointly regulated by Dorsal and STAT, and this regulation is dependent on MAP3K15 (Figs 2I and 3E). The function of *wsv069* (encoding the IE1 protein), has been extensively characterized [20,50–52]. The *wsv249* encodes an E3 ubiquitin ligase that interacts with a shrimp ubiquitin-conjugating enzyme to mediate ubiquitination [53,54]. Silencing *wsv249* resulted in a lower viral load compared to *wsv069* silencing (S5E–S5G Fig), highlighting the importance of the ubiquitination process was important for WSSV invasion. Moreover, the *in vivo* functions of two immediate-early genes, *wsv100* and *wsv107*, were investigated using RNA interference. The results showed that both genes contribute to viral amplification (Fig 3G–3I). Taken together, investigating the transcription mechanism and functions of *ie* genes provides valuable insights into the mechanisms of viral infection and replication, aiding disease control strategies.

The ligands and receptors of the JAK/STAT signaling pathway differ significantly between species. In *Drosophila melanogaster*, the JAK/STAT pathway includes three ligands (Upd1, Upd2, and Upd3), one membrane receptor (Domeless), a short receptor (CG14225/latran), one JAK (hopscotch), and one STAT (stat92E) [55–59] Similarly, aquatic shrimp possess one membrane receptor (Domeless), one JAK, and one STAT (analogous to mammalian STAT5), and only two ligands have been reported until now (CC-CL and peroxiredoxin4, Prx4) [43,44,60,61]. The binding of CC-CL or Prx4 to Domeless activates the JAK/STAT pathway, inducing antimicrobial peptide expression and enhancing antibacterial immunity in shrimp [43,44]. However, whether the same mechanism is at work during viral infection remains unknown. Our results demonstrate that CC-CL is constitutively expressed in the hemolymph of several penaeid shrimp species (Fig 4H). Upon WSSV infection, its transcription is upregulated through the nuclear translocation of the MAP3K15/Dorsal complex. The concentration of CC-CL in the hemolymph increases over the course of infection (Fig 4F). Functioning as a cytokine, CC-CL activates the Domeless-JAK/STAT-*ie* signaling cascade subsequently, facilitating viral replication. Silencing CC-CL reduced viral gene expression, while CC-CL overexpression enhanced STAT-regulated *ie* genes expression (Fig 5). To our knowledge, this is the first report on the expression mechanism of CC-CL and its mediating between the NF-κB and JAK/STAT pathways during viral infection.

Interestingly, while both CC-CL and Prx4 contribute to antibacterial immunity in shrimp by interacting with Domeless and activating the JAK/STAT pathway, their functions in response to viral invasion are quite distinct. CC-CL promotes viral amplification (Fig 5), whereas Prx4 exhibits an antiviral function *in vivo* [62]. The pulldown assay showed that the interleukin 6 receptor (ILR) alpha domain in the shrimp Domeless is the interaction region for CC-CL, while Domeless binding with Prx4 requires a larger sequence region (the CBM domain) and the cysteine residues of Prx4 [43,44]. This may partially explain the functional differences observed between CC-CL and Prx4. However, the underlying mechanisms for this difference still require further investigation.

Phosphorylation of MAP3K is a critical step in the MAPK signaling pathway. Among MAP3K family members, the amino acid sequences of the activation segments in their kinase domains are highly conserved (S2 and S3A Figs). Our study found that WSSV infection triggers phosphorylation of MAP3K15 at a very early time (Fig 6A) while the underlying mechanism remains to be elucidated. Treatment with SDK1 inhibited MAP3K15 phosphorylation *in vivo* (Fig 6C), thereby reducing MAP3K15-Dorsal interactions, attenuating the Dorsal-CC-CL-STAT-IE axis (Fig 7), and suppressing the MAP3K15-JNK/P38-c-Jun-IE1 pathway (Fig 8). These effects collectively inhibited viral amplification. Furthermore, SDK1 exhibited similar protective effects against WSSV infection in other crustaceans, including *M. japonicus*, *P. clarkii*, and *P. trituberculatus* (Fig 6G–6I). Adding SDK1 to water improved larval survival rates, suggesting its broad-spectrum inhibitory potential in crustaceans and promising application in aquaculture (S7G–S7I Fig).

In conclusion, MAP3K15 is a promising target for preventing WSSV infections in crustaceans. This study provides a theoretical foundation and novel perspectives for the development of antiviral drugs tailored to economically important crustacean aquaculture species.

## Materials and methods

### Ethics statement

All animal experiments were approved by the Institutional Animal Care and Use Committee (IACUC) of the School of Life Sciences, Shandong University (protocol number SYDWLL-2023–077). All procedures were carried out in strict accordance with national and institutional guidelines for the care and use of laboratory animals.

### Animals and white spot syndrome virus (WSSV)

Healthy shrimp *Marsupenaeus japonicus*: adult (5–10 g) or postlarvae (0.5 cm); *Litopenaeus vannamei*: adult (8–12 g); Crab *Portunus trituberculatus*: adult (250 g); were obtained from a farm in Jimo District, Qingdao City, Shandong Province, China, cultured in a circulating mariculture system at temperatures of about 20–23°C and salinity of about 22‰ (w/v). Crayfish *Procambarus clarkii*: adult (5–10 g) obtained from a breeding farm in Huai ‘an Jiangsu Province, China, were cultured at room temperature in the laboratory. Animals were fed with a commercial diet daily and were randomly selected for study. The animal-related experiments were performed with the approval of the Animal Ethical Committee of Shandong University School of Life Sciences.

The WSSV inoculum was prepared as described in the previous study [63]. For infection assay, the WSSV inoculum was diluted to 5 × 10^5^ with PBS, and each shrimp was injected with 20 μL, the same volume of sterile phosphate-buffered saline (PBS: 140 mM NaCl, 2.7 mM KCl, 10 mM Na_2_HPO4, 1.8 mM KH_2_PO4, pH 7.4) was injected as control.

### RNA extraction, cDNA synthesis, and quantitative real-time PCR (qPCR)

Each of the samples was extracted using tissues from 3–5 shrimp and all the experiments were performed three times independently. Total RNA was extracted from the different tissues of three shrimp using TRIzol (ET101, Transgen, Beijing, China). First-strand cDNA synthesis was performed using a cDNA Synthesis Kit (ABScript III RT Master Mix for qPCR with gDNA Remover, RK20429, Wuhan Abclonal Technology), following the manufacturer’s instructions. The 10-fold diluted cDNAs were used as the templates for qPCR. The reaction mixture consisted of 5 μL of the 2X Universal SYBR Green Fast qPCR Mix (RK21203, Wuhan Abclonal Technology), 1 μL of 1:10 diluted cDNA, and 2 μL of forward and reverse primers (0.5 μM each). qPCR was performed using the CFX96 Real-Time System (Bio-Rad) with the following reaction conditions: 95°C for 3 min; 40 cycles of 95°C for 5 s and 60°C for 30 s; and melting curve analysis was performed from 68°C to 95°C. Primers used are listed in the S1 Table in the supplemental material. For gene expression analysis, *β*-actin was detected simultaneously as the internal reference. The qPCR data were processed using the 2^−ΔΔ^CT method [64].

### Western blot

Shrimp tissue proteins were extracted using Cell lysis buffer for Western and IP (P0013, Beyotime, Shanghai, China). According to the test requirements, 100 mM PMSF (ST506, Beyotime, Shanghai, China), Protease Inhibitor Cocktail (K1007, APExBIO, America), and Phosphatase Inhibitor Cocktail (M7528, AbMole, America) were added according to the volume ratio of 1:100. The protein concentration was determined using a Bradford protein assay kit (C503031, Sangon Biotech, Shanghai, China). The protein samples were added with 1/4 volume of 5 × Sample Loading Buffer (epizyme, LT101). The mixture was boiled at 100°C for 10 min and centrifuged again, and the supernatants were separated by 7.5% or 12.5% SDS-PAGE. After electrophoresis, the proteins were transferred onto a nitrocellulose membrane using transfer buffer (48 mM Tris–HCl, 39 mM glycine, 1.28 mM SDS, and 20% ethanol) for Western blot. The membrane was blocked with 5% nonfat milk and incubated with the corresponding primary antibodies overnight at 4°C or 4 h at room temperature. After washing three times with TBST (0.1% Tween-20 added to TBS), the membranes were incubated with AP-alkaline phosphatase secondary antibodies or HRP-conjugated secondary antibodies for 2 h at room temperature with gentle shaking. After washing three times with TBST, the membrane was placed in a mixture of 10 ml TBS, 45 μL NBT (0.075 g/ml, 70% DMF, 30% DEPC water), 35 μL BCIP (0.05 g/ml, DMF) in dark. For the membrane that used HRP-conjugated secondary antibodies, the signals of protein were visualized using the High-sig ECL western blot substrate (Tanon, Shanghai, China; 180–5001), and captured using a Tanon 5200 Chemiluminescence Imaging System. The intensities of immunoreactive protein bands were quantified using ImageJ software (NIH, Bethesda, MD, USA).

### Antibodies

Phospho-MAP3K5-T918 Rabbit pAb (AP0061), Phospho-P38 MAPK-T180/Y182 Rabbit pAb (AP0526), Phospho-JNK1/2-T183/Y185 Rabbit pAb (AP0473), Phospho-STAT5-Y694 Rabbit pAb (AP0887) and HRP Goat Anti-Rabbit IgG (H + L) (AS014) were purchased from Wuhan Abclonal Technology. Histone-H3 Polyclonal antibody (17168–1-AP) was obtained from Wuhan Proteintech. AP, Goat Anti-Rabbit IgG (A21120) was provided by Wuhan Abbkine. Antibodies were used following the manufacturer’s instructions. Anti-MAP3K15, anti-Dorsal, anti-STAT, anti-c-Jun, anti-FOXO, anti-VP28, and anti-CC-CL, anti-Prx1, anti-Prx4, anti-P38, anti-JNK, and anti-*β*-Actin rabbit pAbs were generated and stored in our laboratory and were used at 1:500–1:1000 dilutions.

### RNA interference (RNAi) assay

RNAi was performed to specifically knock down the expression of the target gene by application of double-stranded RNA (dsRNA) *in vivo*. The partial DNA fragments of target genes were amplified using specific primer pairs containing the T7 promoter sequence (S1 Table) and used as the template for dsRNA synthesis, and dsRNA was synthesized according to the manufacturer’s instructions MEGAscript RNAi Kit (AM1626, Thermofisher scientific). The dsRNA specific for the green fluorescent protein (GFP) gene was produced as a control. The dsRNA was injected into shrimp abdominal at a dose of 5 μg/g shrimp. The same dose was given again 12 hours later. 24 h after the second injection, RNA or protein was extracted from shrimp tissue, and then RNAi efficiency was analyzed using qPCR or western blot. Three shrimp were randomly selected for each experiment to eliminate individual differences.

### Quantification of viral titers

The WSSV *vp28* gene fragment was cloned into a pBlueScript vector. The plasmid sample, for which the copy number had been calculated, was serially diluted. Genomic DNA was extracted from the viral inoculum (100 μL) or tissue (10 mg) using MagExtractor Genome (Toyobo, Osaka, Japan; NPK-101). The genomic DNA was extracted from the intestine homogenate using MagExtractor Genome (Toyobo; NPK-101), according to the manufacturer’s instructions. DNA concentration was determined using a NanoDrop 2000 spectrophotometer (Thermo Fisher Scientific, Waltham, MA, USA). qPCR was performed. Primers used are listed in the S1 Table in the supplemental material. The standard curve generated using the data of plasmid samples was used to quantify the viral titer in the viral inoculum or infected tissues.

### Recombinant expression, purification, and antiserum production

Specific primers (S1 Table) were used to amplify fragments encoding MAP3K15, and CC-CL, which were ligated into the pET30a (+) vector. The recombinant plasmid was transformed into *Escherichia coli* Rosetta (DE3) strain for expression under induction by 0.5 mM isopropyl-*β*-D-thiogalactopyranoside at 37°C for 4-6 h.

Recombinant proteins were purified using Ni Sepharose 6 Fast Flow (17531801, Cytiva, America). The resin was fully washed with cold 0.1% Triton X-114, which could reduce endotoxin contamination to less than 3 EU/mg, before the final elution of proteins using imidazole. The resultant proteins were dialyzed thoroughly in PBS, concentrated to 1-1.5 mg/mL, and washed with cold 0.1% Triton X-114, which could reduce endotoxin contamination to less than 0.3 EU/mg  and stored at -80°C for use.

Purified recombinant protein of MAP3K15 or CC-CL (1-1.5 mg/ml, 1 ml) was mixed with an equal volume of complete Freund’s adjuvant (F5881; Sigma-Aldrich, St. Louis, MO, USA), emulsified, and used to immunize male New Zealand white rabbits. Immunization was repeated 25 days later using recombinant protein mixed with incomplete adjuvant (F5506; Sigma-Aldrich). Blood was collected to detect the specificity 7 days after the second immunization. For the third time, rabbits were given booster injections of antigen without adjuvant. The rabbit antiserum was collected 7 days later and was used for western blot, and then stored at -80°C. Other antisera were prepared similarly.

### Cytoplasm, nuclear protein extraction

The intestine was dissected from the shrimp and washed three times with PBS. Protein extraction was performed using the cytoplasmic protein extraction kit (C510005-0050, Sangon Biotech, Shanghai, China) and the nuclear protein extraction kit (C500009, Sangon Biotech, Shanghai, China) according to the manufacturer’s instructions. At least four shrimp were used for each sample to eliminate individual differences. The protein concentration was determined using a Bradford protein assay kit (C503031, Sangon Biotech, Shanghai, China).

### Identification of MAP3K15-interacting proteins

Approximately 175 mg of intestine tissue was collected from shrimp under normal conditions or 24 h post-WSSV infection (10 individuals per group). The intestine tissues were homogenized in IP buffer (P0013, Beyotime, Shanghai, China), and the homogenates were centrifuged at 4°C. The resulting supernatant (800 µL, 1.5 mg/mL) was incubated with 50 µL of Protein A beads (L00210-10, GenScript, Nanjing, China) at 4°C for 30 min to eliminate nonspecific binding. After centrifugation at 4°C for 3 min, the supernatant was collected and incubated with 80 µL of anti-MAP3K15 antibody at 4°C overnight. Subsequently, 50 µL of Protein A beads was added, and the mixture was incubated at 4°C for 2 h. The beads were collected, washed three times with PBS, and analyzed by SDS-PAGE. The protein bands containing potential MAP3K15 interacting proteins were excised and delivered to Shanghai Bioprofile Company (SBC) for LC-MS/MS analysis. All procedures and methods used in the LC-MS/MS followed the standard guidelines of SBC. The MS data were analyzed using MaxQuant software version 1.5.8.3. MS data were searched against database retrieval uniprot-Penaeus (https://www.uniprot.org/taxonomy/27405). The database search results were filtered and exported with <1% false discovery rate (FDR) at peptide-spectrum-matched level, and protein level, respectively.

### Co-immunoprecipitation (Co-IP)

Co-IP was performed to confirm the interaction between MAP3K15 and Dorsal *in vivo*. The intestine from WSSV-infected shrimp was lysed in IP buffer (P0013, Beyotime, Shanghai, China). The lysate was centrifuged at 4°C, 12000 × g for 15 min, and the obtained supernatant (about 500 μL, 1 mg/mL) was used as an IP protein pool. Add 30 μL Protein A beads (L00210-10, GenScript, Nanjing, China) into the supernatant, and gently incubate at 4°C for 30 min to remove non-specific binding. Then centrifuged at 4°C, 500 × g for 2 min, added the corresponding antibody (MAP3K15 and Dorsal) to the supernatant, and incubated at 4°C overnight. Protein A beads were added to capture the antibodies for 2 h with agitation at 4°C. After washing with PBS three times, the immunoprecipitates were eluted by boiling the beads in 1% SDS sample buffer and then detected by western blot.

### Immunoprecipitation of CC-CL protein from shrimp hemolymph

Shrimp hemolymph were collected at 0, 6 and 24 h post-WSSV infection by centrifugation at 800 × g for 5 min at 4°C, and the supernatant was collected. Hemolymph was then clarified by centrifugation at 12 000 × g for 10 min at 4°C. To deplete nonspecific binding, 50 µL Protein A beads was added to the hemolymph and incubated for 30 min at 4°C, followed by centrifugation at 500 × g for 2 min; the unbound fraction was retained. The pre-cleared hemolymph was incubated overnight at 4°C with 50 µL anti-CC-CL antibody, then with an additional 50 µL Protein A beads for 2 h at 4°C to capture immune complexes. Beads were washed three times with ice-cold PBS, resuspended in SDS–PAGE sample buffer, and heated at 95°C for 10 min. Immunoprecipitated proteins were analyzed by western blot.

### Fluorescent labeling of MAP3K15 and Dorsal antibodies

MAP3K15 antibody (2 mg/mL, 1 mg) was conjugated with FITC following the kit instructions (ARL0021K, Frdbio, Wuhan, China). The antibody was loaded into a centrifugal filter unit, mixed with 1/10 volume of labeling buffer, and centrifuged at 12 000 × g for 10 min at 4°C. This buffer-exchange step was repeated three times to remove low-molecular-weight contaminants, yielding an antibody concentration of ~2 mg/mL. Next, 50 µL of FITC working solution was added to the filter unit and incubated at 37°C in the dark for 60 min. After incubation, added with 1/10 volume of FITC removal reagent, and the mixture was centrifuged again at 12 000 × g for 10 min at 4°C to remove unbound dye. The retentate was washed with labeling buffer until the filtrate remained colorless, and the purified FITC-conjugated MAP3K15 antibody was recovered from the filter unit.

Dorsal antibody (2 mg/mL, 1 mg) was conjugated with Fluor647 following the kit instructions (ARL0560, Frdbio, Wuhan, China). The antibody was first mixed with one-tenth volume of labeling buffer, then combined with 200 µL activated Fluor647 (antibody: dye molar ratio 1:23), gently mixed, and incubated at 37°C in the dark for 1 h. Upon completion, approximately 450 µL PBS was added and the mixture was transferred to an ultrafiltration tube, which was centrifuged at for 5 min at 4°C to remove unbound dye. The antibody was washed with an equal volume of PBS at least five times until the filtrate was clear, and the Fluor647-labeled Dorsal antibody was recovered from the filter unit.

### 3D structural modeling of the MAP3K15-Dorsal heterodimer

The 3D protein structure of Dorsal was obtained from the AlphaFold (https://alphafold.ebi.ac.uk/) Protein Structure Database, while that of MAP3K15 was predicted using SWISS-MODEL (https://swissmodel.expasy.org/). The residue/interactions of protein complexes were predicted using the HDOCK web server (http://hdock.phys.hust.edu.cn/). The predicted results were visualized by PyMol (https://pymol.org/2/).

### Immunocytochemistry assay

The hemocytes were collected from the ventral sinus using a syringe with cold anticoagulant buffer 1ml (0.45 M NaCl, 10 mM KCl, 10 mM EDTA, and 10 mM HEPES, pH 7.45) containing 4% paraformaldehyde, fixed for 10 min, and then immediately centrifuged at 800 × g for 5 min at 4°C to obtain the hemocytes. Then the hemocytes were washed twice times with PBS and fixed with 4% paraformaldehyde for 30 min. Subsequently, the hemocytes were spread onto poly L-lysine-coated glass slides and washed three times with PBS. After incubation with 0.2% Triton X-100 in PBS for 10–15 min, the cells were washed three times with PBS, blocked with 3% bovine serum albumin (BSA) in PBS at 37°C for 30 min, and incubated with the primary antibodies (anti-Dorsal, anti-STAT, anti-c-Jun, 1:100 diluted in 3% BSA) overnight at 4°C. The hemocytes were washed with PBS three times, incubated with the goat anti-rabbit Alexa Fluor 488 antibodies (A23220, Abbkine, Wuhan, China) for 2 h at 37°C, washed with PBS again, and then stained with 4–6-diamidino 2-phenylindole (DAPI) (AS-83210, AnaSpec, San Jose, CA, USA) for 10 min at room temperature. After washing three times, the slides were examined under a fluorescent microscope (Olympus BX51, Japan). Immunofluorescence images were analyzed using ImageJ software (NIH, USA) to assess the nuclear localization of Dorsal and STAT. Each experimental group included three independent biological replicates. For each replicate, no fewer than three random fields were selected for image acquisition and analysis. Nuclear localization was quantified by calculating the nuclear-to-cytoplasmic fluorescence intensity ratio of the target proteins, reflecting their relative distribution within the nucleus.

### Chromatin immunoprecipitation (ChIP)

ChIP assays were conducted to examine the binding of Dorsal and STAT to *ie* gene promoters following MAP3K15 knockdown or inhibition. Promoter binding sites of the target genes were predicted using the JASPAR database (http://jaspardev.genereg.net/), and gene-specific primers were designed accordingly (S1 Table). Hemocytes were collected at 6 hours post-WSSV injection and pooled for ChIP analysis. The assay was performed using a ChIP Assay Kit (P2078, Beyotime, Shanghai, China) following the manufacturer’s instructions. Immunoprecipitated DNA was analyzed by qPCR using specific primers (S1 Table) targeting fragments containing Dorsal and STAT5 binding sites. Fold enrichment was calculated to assess binding activity.

### Generation and *in vivo* application of recombinant CC-CL

The full-length fragment of CC-CL was cloned into plasmid pET30a and transformed into Rosetta (DE3) for recombinant expression, the primers are shown in S1 Table. The recombinant protein was expressed as a soluble protein. The protein tags generated by the empty pET30a plasmid were prepared simultaneously as controls. The majority of endotoxin contamination was removed by thorough washing using cold 0.1% Triton X-114. The protein concentration was determined using a Bradford protein assay kit (C503031, Sangon Biotech, Shanghai, China). rCC-CL or the His-tag was injected into shrimp with the WSSV inoculum with a dose of 5 μg/g per shrimp.

### Inhibitor application

SDK1 was dissolved in water containing 5% dimethyl sulfoxide (DMSO), diluted to the indicated concentration with PBS, and administered *in vivo* at a dose of 0.5 μg/g shrimp. The PBS-5% DMSO solvent was used as the control. The inhibitor was administered 2 hours before the follow-up experiment.

### Simulate natural WSSV infection

About 300 postlarvae were divided evenly into three groups (Normal, PBS + WSSV, SDK1 + WSSV). Add 200 μL of WSSV (1 × 10^5^) to approximately 3 L seawater to simulate the environment of natural WSSV infection. At the same time, inhibitor SDK1 (final concentration about 40 nM) was added to seawater.After 2 days of WSSV immersion, about 20 postlarvae were randomly selected, and virus genes *ie* *1* and *vp28* were detected by qPCR. Meanwhile, the dead postlarvae were recorded every 24 hours for 7 days.

### Survival rate assay

Shrimp were randomly assigned to two or three groups (n ≥ 30 per group). At 24 hours after the second injection of either *dsMAP3K15* or control *dsGFP*, or following injection of the inhibitor (SDK1) or mock solution (PBS with 5% DMSO), each shrimp in both the experimental and control groups was injected with WSSV (1 × 10⁷ copies) or PBS. Survival was monitored by counting the number of live shrimp every 12 or 24 hours.

### Statistical analysis

All experiments included at least three biological replicates (n ≥ 3) to ensure the reliability and reproducibility of the results, in accordance with the principles of proper experimental design. Western blot bands were quantitatively analyzed using ImageJ software, and the results are presented as relative band intensities normalized to the control group (set to 1). Survival analysis was performed using the Log-rank (Mantel-Cox) test. Bar graphs are expressed as the mean ± standard deviation (SD). Statistical significance was assessed using a two-tailed Student’s *t*-test with GraphPad Prism software (GraphPad Inc., La Jolla, CA, USA). Significance levels were defined as follows: ns, not significant; *, *P* < 0.05; **, *P *< 0.01; ***, *P* < 0.001; ****, *P *< 0.0001.

## Supporting information

S1 Fig Expression pattern and functional analysis of MAP3K15 during WSSV infection. (A-C) Temporal expression profiles of MAP3K15 in shrimp hemocytes (A), gills (B) and intestine (C) at 0, 2, 6, 12, 24, 48 and 72 (hpi) with WSSV or PBS, as assessed by qPCR. Data are presented as mean ± SD. Statistical comparisons were performed by two-way ANOVA with multiple comparisons. Groups sharing the same letter are not significantly different, whereas groups bearing different letters differ significantly. (D) The recombinant expression and purification of the S_TKC domain of MAP3K15 in *Escherichia coli* (*E. coli*). Lane 1: total proteins with MAP3K15-pET30a; Lane 2: total proteins after IPTG induction; Lane 3: purified recombinant MAP3K15. M, Protein molecular mass markers. (E) The polyclonal antibody of *Mj*MAP3K15 was utilized to detect the recombinant MAP3K15 and MAP3K15 protein in shrimp tissue (intestines). M, Protein molecular mass markers. (F) Western blot analysis of MAP3K15 expression in shrimp hemocytes at various time points post-WSSV infection. (G) RNAi efficiency of MAP3K15 knockdown in shrimp hemocytes was analyzed by qPCR. (H) Expression of *ie* *1* and *vp28* at different time points in hemocytes of MAP3K15-knockdown and *dsGFP* shrimp were analyzed by qPCR. (I) Cumulative survival curves of shrimp injected with *dsMAP3K15* or *dsGFP* (n = 30 per group) were analyzed by the Log‐rank (Mantel–Cox) test. Data are presented as the mean ± SD from three independent replicates and were analyzed by Student’s *t*-test. *, *P* < 0.05, **, *P *< 0.01, ***, *P* < 0.001. *β*-Actin served as the internal reference for all qPCR and western blot analyses; relative expression values were normalized to the control group (set to 1).
(TIF)

S2 Fig Sequence alignment of MAP3K15 and ASK1. The MAP3K15 and ASK1 sequences of several species were derived from GenBank. The black box area is the Pfam: DUF4071 domain. The area in the red box is the S_TKC domain.
(TIF)

S3 Fig Domain architecture and phylogenetic tree of MAP3K15 and ASK1. (A) The domain architecture of MAP3K15 from different species (*Homo sapiens*, *Mus musculus*, *Drosophil*a, and *Marsupenaeus japonicus*) was analyzed using SMART software (http://smart.embl-heidelberg.de/). The numbers show the starting and ending residues of a module and the total length of the sequence. aa, amino acids. (B) Phylogenetic trees of MAP3K15 and ASK1 from different species were constructed using MEGA 11.0 with 1,000 bootstrap replicates. The MAP3K15 of *M*. *japonicus* was denoted by a red star.
(TIF)

S4 Fig Subcellular distribution of Dorsal in shrimp hemocytes following MAP3K15 knockdown. (A) The immunoprecipitated proteins were separated by SDS-PAGE and then stained by Coomassie bright blue. (B) Subcellular localization of Dorsal in shrimp hemocytes was visualized by immunocytochemistry following pretreatment with either *dsGFP* or *dsMAP3K15*. Scale bars = 20 μm. (b) The nuclear-to-cytoplasmic fluorescence intensity ratio of Dorsal was quantitatively analyzed using ImageJ software. (C) The distribution of Dorsal between cytoplasmic and nuclear fractions in shrimp hemocytes pretreated with *dsGFP* or *dsMAP3K15* was examined by western blot. Data are presented as the mean ± SD from three independent replicates and were analyzed by Student’s *t*-test. ns, no significant difference. Western blot bands were quantified using ImageJ; the resulting values represent relative band intensities normalized to the control group (set to 1).
(TIF)

S5 Fig STAT localization in hemocytes following MAP3K15 knockdown and antiviral effects of *wsv249*/*wsv069* in shrimp. (A) Western blot analysis of STAT and FOXO distribution in cytoplasmic and nuclear fractions isolated from shrimp intestine following pretreatment with *dsGFP* or *dsMAP3K15*. (B) Immunocytochemical detection of STAT nuclear translocation in hemocytes. Scale bars = 20 μm. (b) The nuclear-to-cytoplasmic fluorescence intensity ratio of STAT was quantitatively analyzed using ImageJ software. (C) Western blot analysis of phosphorylated STAT (p-STAT) in intestinal lysates from shrimp pretreated with *dsGFP* or *dsMAP3K15*. (D) Schematic representation of the experimental workflow. (E) qPCR quantification of RNAi knockdown efficiency for *wsv249* and *wsv069*, and relative *vp28* transcript levels in the intestine at 48 h post-WSSV challenge. (F) WSSV genome copy numbers in intestinal tissue of *wsv249*-, *wsv069*-knockdown, and *dsGFP* control shrimp, determined by qPCR. (G) Western blot detection of VP28 protein levels in the intestine after *wsv249* or *wsv069* knockdown. Data are presented as the mean ± SD from three independent replicates and were analyzed by Student’s *t*-test. ns, no significant difference, **, *P *< 0.01. ***, *P *< 0.001. *β*-actin served as the internal reference for all the qPCR. Western blot bands were quantified using ImageJ. The numbers indicate the relative band intensities. *β*-Actin, Histone was used as an internal reference.
(TIF)

S6 Fig CC-CL expression, gene silencing efficiency, and recombinant protein detection in shrimp. (A) The expression profiles of CC-CL in intestine at 0, 2, 6, 12, and 24 hpi were analyzed by qPCR. (B) RNAi efficiency of CC-CL knockdown in shrimp intestine detected by qPCR. (C) Recombinant expression and purification of CC-CL in *E. coli*. Lane 1: total proteins with CC-CL-pET30a; Lane 2: total proteins after IPTG induction; Lane 3: purified recombinant CC-CL (rCC-CL). M, Protein molecular mass markers. Data are presented as the mean ± SD from three independent replicates and were analyzed by Student’s *t*-test. ns, no significant difference, *, *P* < 0.05, **, *P *< 0.01. *β*-actin served as the internal reference for all the qPCR analyses.
(TIF)

S7 Fig SDK1 suppresses WSSV replication and improves shrimp survival. (A) Expression levels of *ie1* and *vp28* in hemocytes at 2, 6, 24, and 48 hpi were analyzed by qPCR. Shrimp were pretreated with SDK1 for 2 h. (B, C) qPCR analysis of *ie1* expression at 6 hpi and *vp28* transcription at 24 hpi in the intestine of *L*. *vannamei*, *P*. *clarkii*, and *P*. *trituberculatus* following 2 h of SDK1 pretreatment and WSSV challenge. (D-F) Survival rates of *M. japonicus* (D), *L. vannamei* (E), and *P. clarkii* (F) following SDK1 treatment, with PBS injection as the control. (n = 30 per group). (G) After 2 days of WSSV immersion, about 20 postlarvae of *M*. *japonicus* were selected and the transcription of *ie* *1* and *vp28* were detected by qPCR. (H) Survival rates of postlarvae treated with SDK1 following WSSV immersion (80 shrimp per group). (I) Images of live postlarvae on the seventh day after WSSV immersion. Data are presented as the mean ± SD from three independent replicates and were analyzed by Student’s *t*-test. ns, no significant difference, **, *P *< 0.01. ***, *P* < 0.001. *β*-actin was used as the internal reference for all the qPCR analyses. Survival rates were analyzed by the Log-rank (Mantel-Cox) test.
(TIF)

S8 Fig SDK1 disrupts MAPK15–Dorsal interaction and inhibits CC-CL expression and STAT activation in *L. vannamei.* (A) Co-immunoprecipitation and western blot of MAP3K15–Dorsal interaction in shrimp cytoplasm at 24 h post-WSSV, following SDK1 or PBS pretreatment. (B-D) Western blot analysis of nuclear STAT and Dorsal levels (B), phosphorylated STAT (C), and CC-CL expression (D) in the intestine of *L. vannamei* following SDK1 treatment and WSSV infection. (E) The expression levels of *wsv249*, *wsv100*, *wsv403*, *wsv107*, and *wsv069* in the intestine of *L. vannamei* were analyzed by qPCR at 6 hours post-WSSV infection following SDK1 treatment. Data are presented as the mean ± SD from three independent replicates and were analyzed by Student’s *t*-test. *, *P *< 0.05. **, *P* < 0.01. *β*-actin was used as the internal reference. Western blot bands were quantified using ImageJ, with values representing relative band intensities.
(TIF)

S1 Table Sequence of primers used in this study. (DOCX)
