# Supplementary figures and images for: MAP3K15 facilitates multiple viral genes expression in crustaceans via Dorsal-CC-CL-STAT axis besides the JNK/P38 pathway
Source: PLoS Pathog. 2025 Aug 1;21(8):e1013349. doi: 10.1371/journal.ppat.1013349 (PMC12316391; doi:10.1371/journal.ppat.1013349)

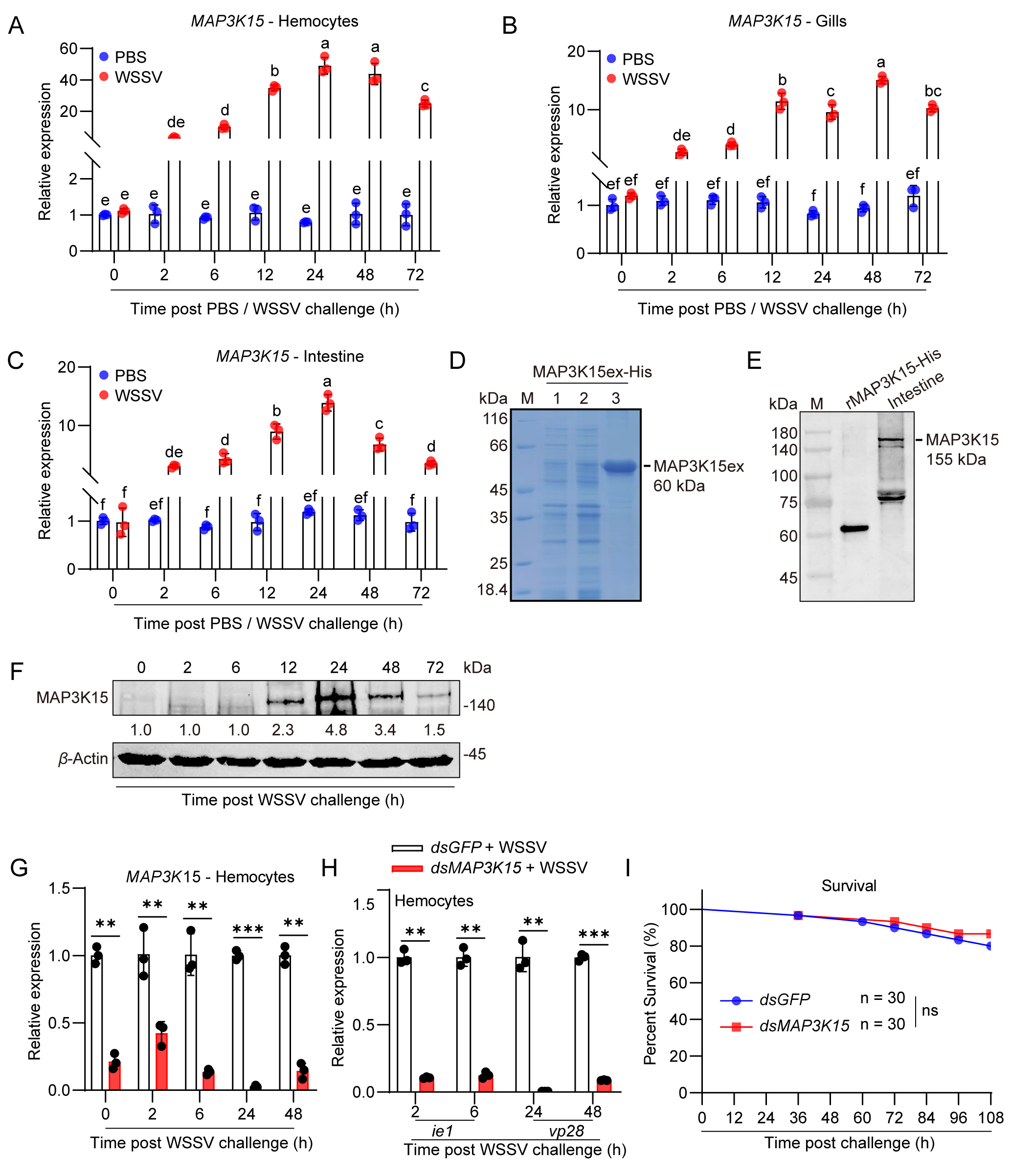

Supplement: S1 Fig — (A-C) Temporal expression profiles of MAP3K15 in shrimp hemocytes (A), gills (B) and intestine (C) at 0, 2, 6, 12, 24, 48 and 72 (hpi) with WSSV or PBS, as assessed by qPCR. Data are presented as mean ± SD. Statistical comparisons were performed by two-way ANOVA with multiple comparisons. Groups sharing the same letter are not significantly different, whereas groups bearing different letters differ significantly. (D) The recombinant expression and purification of the S_TKC domain of MAP3K15 in Escherichia coli (E. coli). Lane 1: total proteins with MAP3K15-pET30a; Lane 2: total proteins after IPTG induction; Lane 3: purified recombinant MAP3K15. M, Protein molecular mass markers. (E) The polyclonal antibody of MjMAP3K15 was utilized to detect the recombinant MAP3K15 and MAP3K15 protein in shrimp tissue (intestines). M, Protein molecular mass markers. (F) Western blot analysis of MAP3K15 expression in shrimp hemocytes at various time points post-WSSV infection. (G) RNAi efficiency of MAP3K15 knockdown in shrimp hemocytes was analyzed by qPCR. (H) Expression of ie 1 and vp28 at different time points in hemocytes of MAP3K15-knockdown and dsGFP shrimp were analyzed by qPCR. (I) Cumulative survival curves of shrimp injected with dsMAP3K15 or dsGFP (n = 30 per group) were analyzed by the Log‐rank (Mantel–Cox) test. Data are presented as the mean ± SD from three independent replicates and were analyzed by Student’s t-test. *, P < 0.05, **, P < 0.01, ***, P < 0.001. β-Actin served as the internal reference for all qPCR and western blot analyses; relative expression values were normalized to the control group (set to 1). (TIF) [file ppat.1013349.s001.tif]

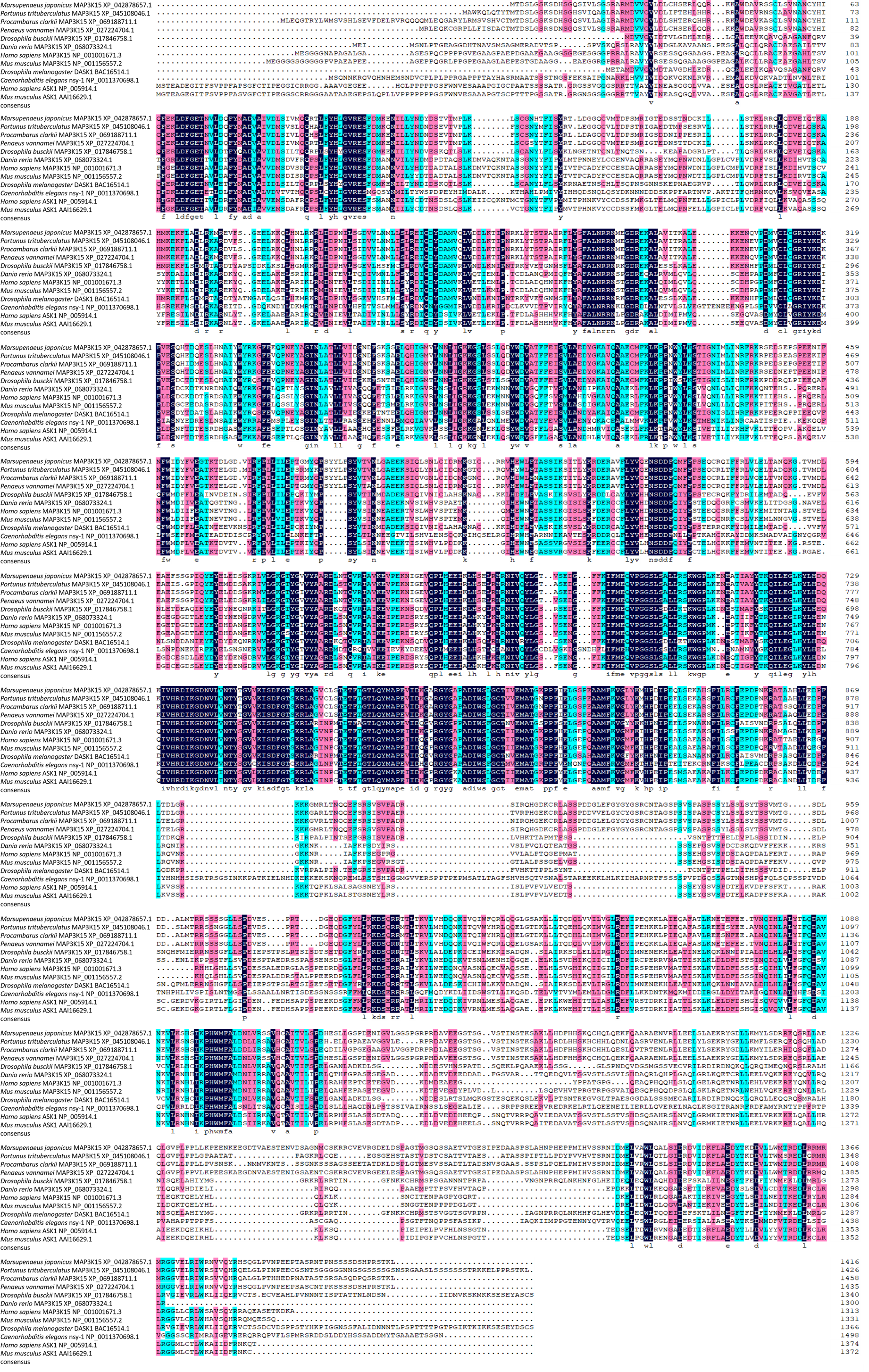

Supplement: S2 Fig — The MAP3K15 and ASK1 sequences of several species were derived from GenBank. The black box area is the Pfam: DUF4071 domain. The area in the red box is the S_TKC domain. (TIF) [file ppat.1013349.s002.tif]

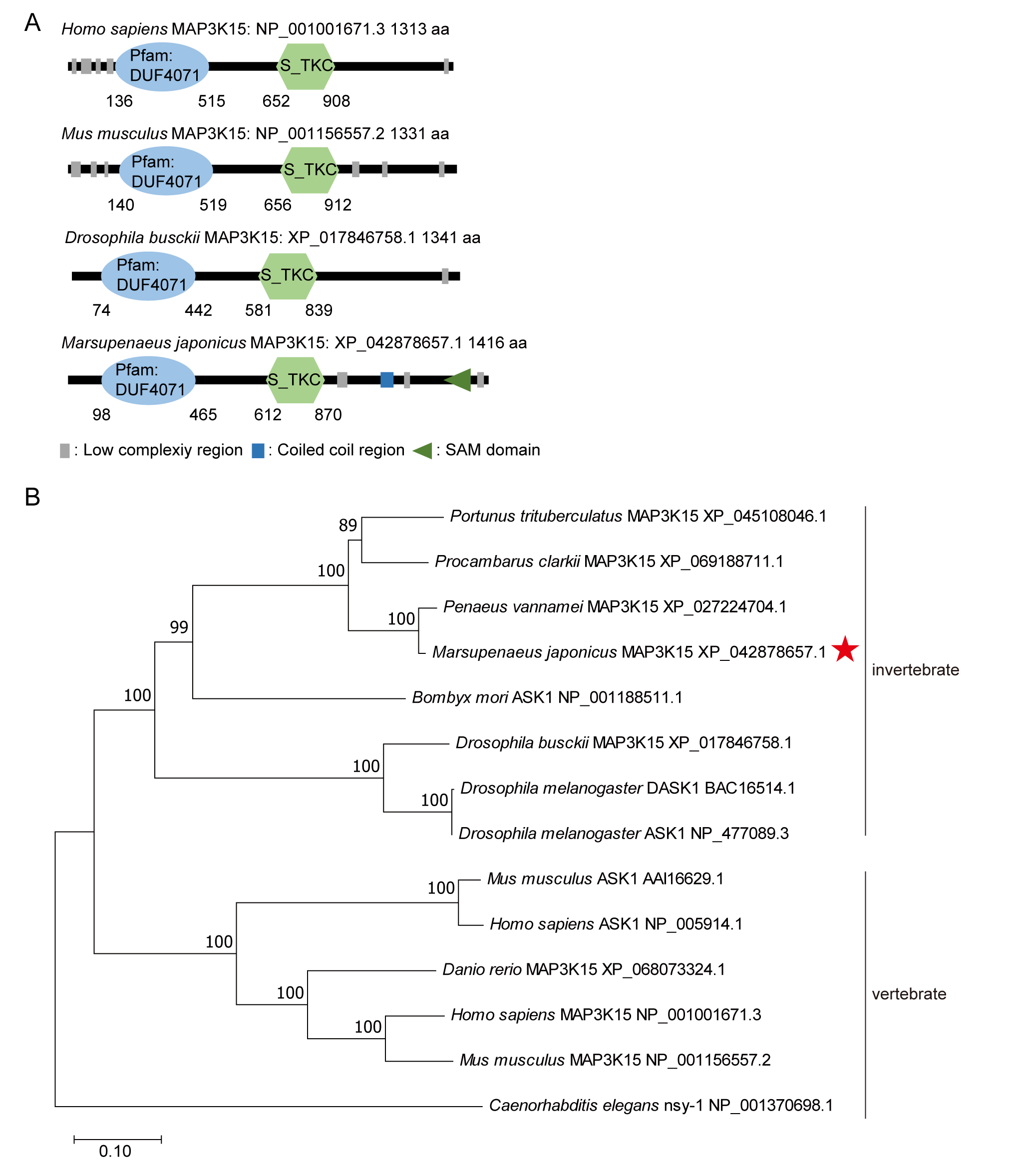

Supplement: S3 Fig — (A) The domain architecture of MAP3K15 from different species (Homo sapiens, Mus musculus, Drosophila, and Marsupenaeus japonicus) was analyzed using SMART software (http://smart.embl-heidelberg.de/). The numbers show the starting and ending residues of a module and the total length of the sequence. aa, amino acids. (B) Phylogenetic trees of MAP3K15 and ASK1 from different species were constructed using MEGA 11.0 with 1,000 bootstrap replicates. The MAP3K15 of M. japonicus was denoted by a red star. (TIF) [file ppat.1013349.s003.tif]

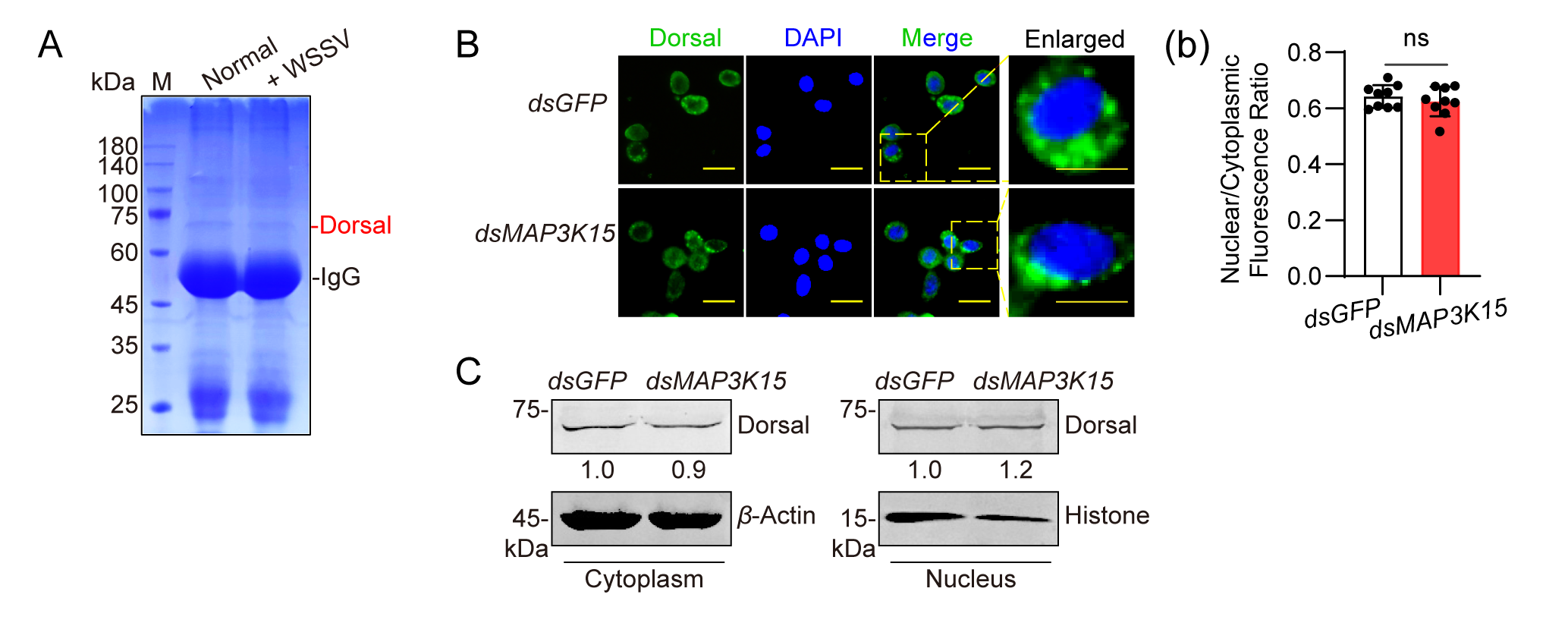

Supplement: S4 Fig — (A) The immunoprecipitated proteins were separated by SDS-PAGE and then stained by Coomassie bright blue. (B) Subcellular localization of Dorsal in shrimp hemocytes was visualized by immunocytochemistry following pretreatment with either dsGFP or dsMAP3K15. Scale bars = 20 μm. (b) The nuclear-to-cytoplasmic fluorescence intensity ratio of Dorsal was quantitatively analyzed using ImageJ software. (C) The distribution of Dorsal between cytoplasmic and nuclear fractions in shrimp hemocytes pretreated with dsGFP or dsMAP3K15 was examined by western blot. Data are presented as the mean ± SD from three independent replicates and were analyzed by Student’s t-test. ns, no significant difference. Western blot bands were quantified using ImageJ; the resulting values represent relative band intensities normalized to the control group (set to 1). (TIF) [file ppat.1013349.s004.tif]

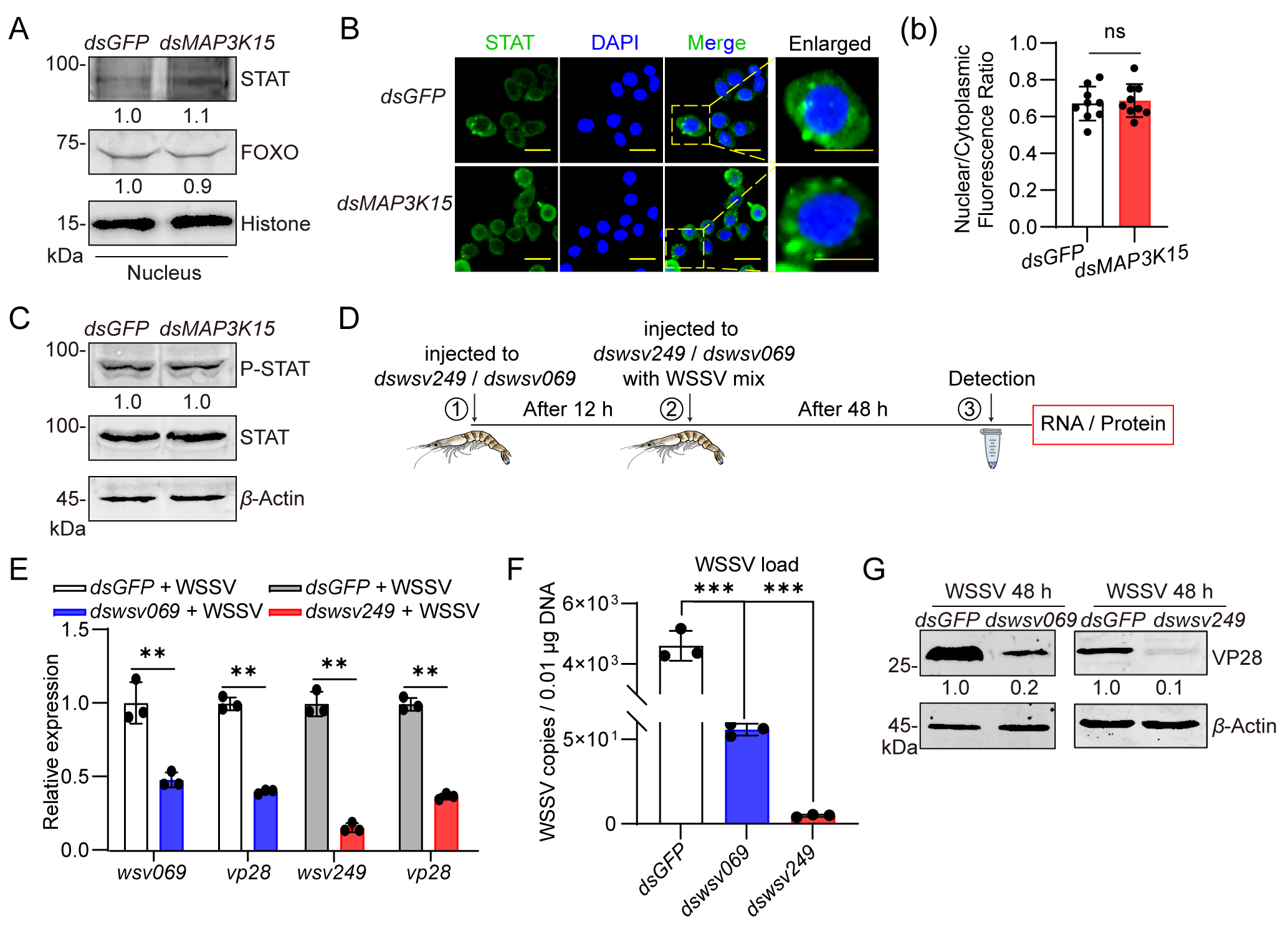

Supplement: S5 Fig — (A) Western blot analysis of STAT and FOXO distribution in cytoplasmic and nuclear fractions isolated from shrimp intestine following pretreatment with dsGFP or dsMAP3K15. (B) Immunocytochemical detection of STAT nuclear translocation in hemocytes. Scale bars = 20 μm. (b) The nuclear-to-cytoplasmic fluorescence intensity ratio of STAT was quantitatively analyzed using ImageJ software. (C) Western blot analysis of phosphorylated STAT (p-STAT) in intestinal lysates from shrimp pretreated with dsGFP or dsMAP3K15. (D) Schematic representation of the experimental workflow. (E) qPCR quantification of RNAi knockdown efficiency for wsv249 and wsv069, and relative vp28 transcript levels in the intestine at 48 h post-WSSV challenge. (F) WSSV genome copy numbers in intestinal tissue of wsv249-, wsv069-knockdown, and dsGFP control shrimp, determined by qPCR. (G) Western blot detection of VP28 protein levels in the intestine after wsv249 or wsv069 knockdown. Data are presented as the mean ± SD from three independent replicates and were analyzed by Student’s t-test. ns, no significant difference, **, P < 0.01. ***, P < 0.001. β-actin served as the internal reference for all the qPCR. Western blot bands were quantified using ImageJ. The numbers indicate the relative band intensities. β-Actin, Histone was used as an internal reference. (TIF) [file ppat.1013349.s005.tif]

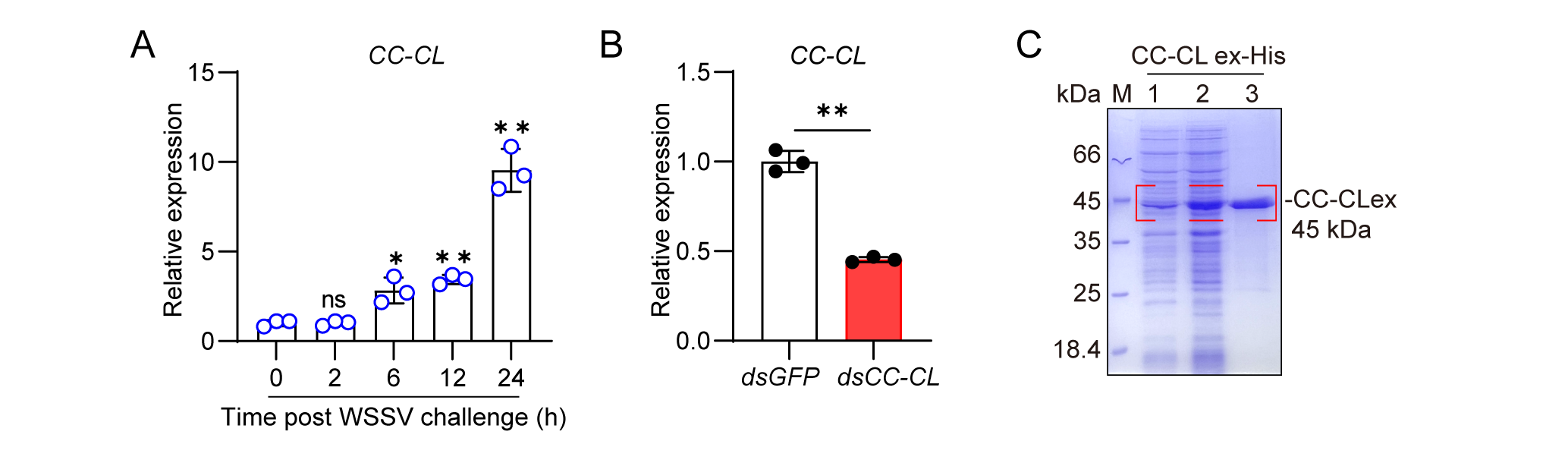

Supplement: S6 Fig — (A) The expression profiles of CC-CL in intestine at 0, 2, 6, 12, and 24 hpi were analyzed by qPCR. (B) RNAi efficiency of CC-CL knockdown in shrimp intestine detected by qPCR. (C) Recombinant expression and purification of CC-CL in E. coli. Lane 1: total proteins with CC-CL-pET30a; Lane 2: total proteins after IPTG induction; Lane 3: purified recombinant CC-CL (rCC-CL). M, Protein molecular mass markers. Data are presented as the mean ± SD from three independent replicates and were analyzed by Student’s t-test. ns, no significant difference, *, P < 0.05, **, P < 0.01. β-actin served as the internal reference for all the qPCR analyses. (TIF) [file ppat.1013349.s006.tif]

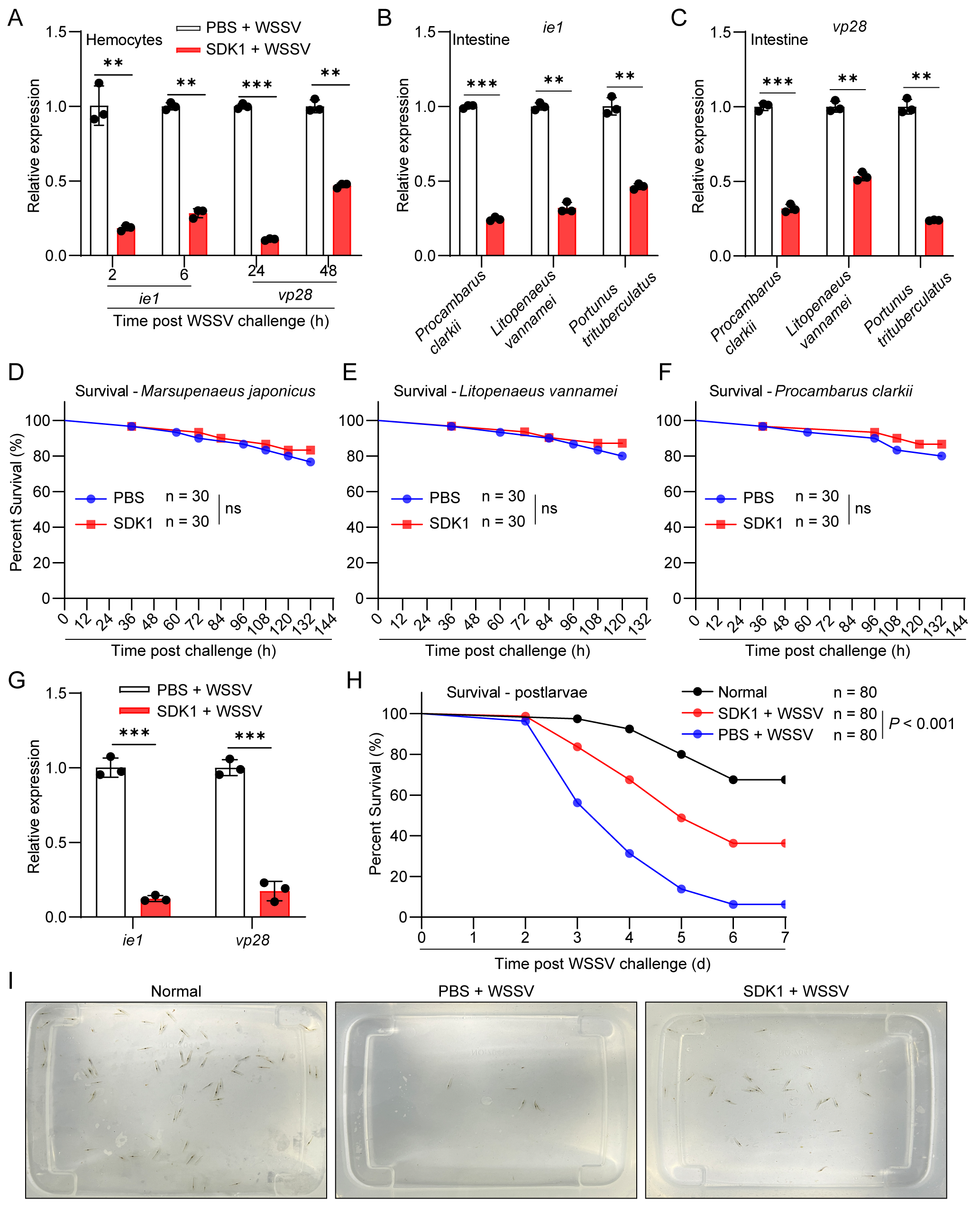

Supplement: S7 Fig — (A) Expression levels of ie1 and vp28 in hemocytes at 2, 6, 24, and 48 hpi were analyzed by qPCR. Shrimp were pretreated with SDK1 for 2 h. (B, C) qPCR analysis of ie1 expression at 6 hpi and vp28 transcription at 24 hpi in the intestine of L. vannamei, P. clarkii, and P. trituberculatus following 2 h of SDK1 pretreatment and WSSV challenge. (D-F) Survival rates of M. japonicus (D), L. vannamei (E), and P. clarkii (F) following SDK1 treatment, with PBS injection as the control. (n = 30 per group). (G) After 2 days of WSSV immersion, about 20 postlarvae of M. japonicus were selected and the transcription of ie 1 and vp28 were detected by qPCR. (H) Survival rates of postlarvae treated with SDK1 following WSSV immersion (80 shrimp per group). (I) Images of live postlarvae on the seventh day after WSSV immersion. Data are presented as the mean ± SD from three independent replicates and were analyzed by Student’s t-test. ns, no significant difference, **, P < 0.01. ***, P < 0.001. β-actin was used as the internal reference for all the qPCR analyses. Survival rates were analyzed by the Log-rank (Mantel-Cox) test. (TIF) [file ppat.1013349.s007.tif]

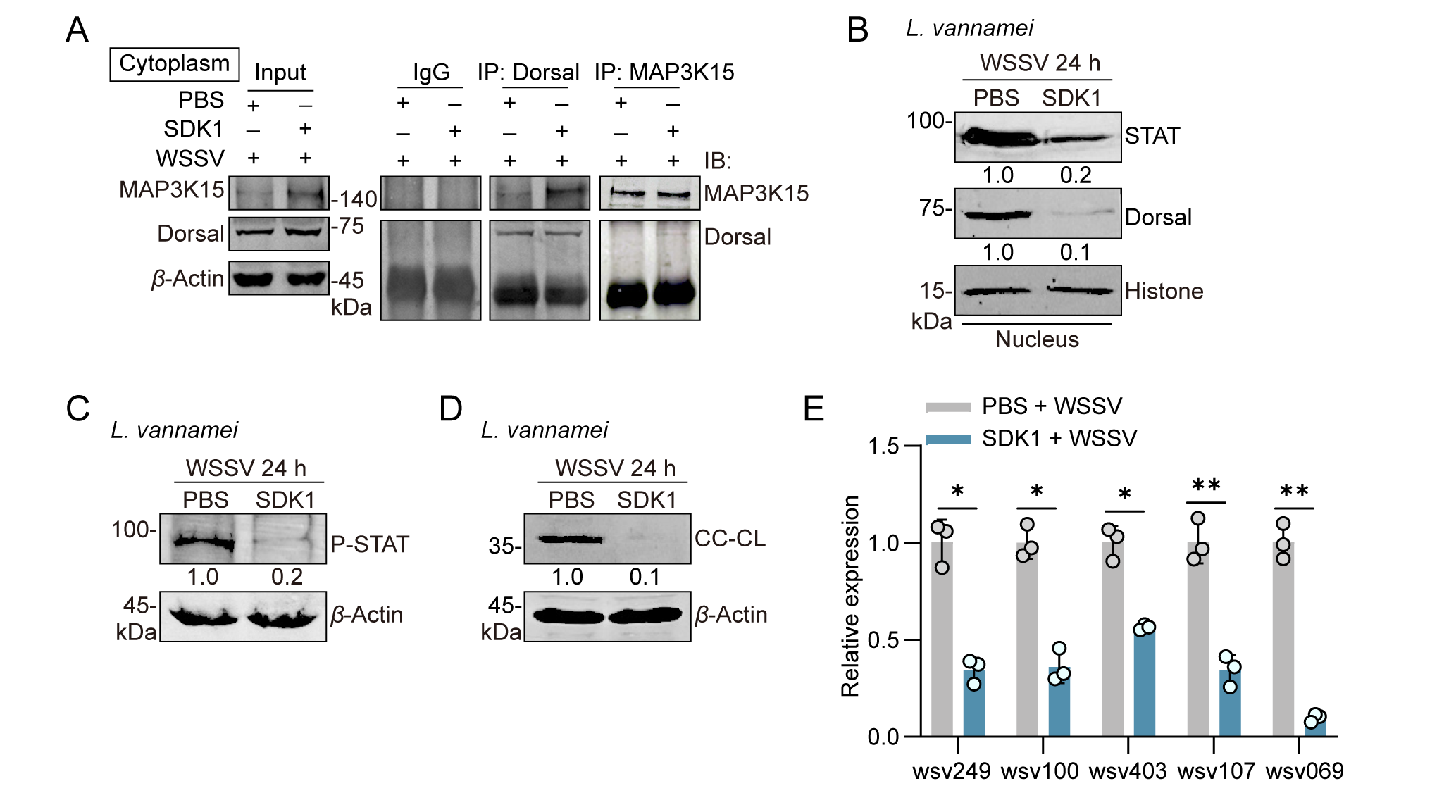

Supplement: S8 Fig — (A) Co-immunoprecipitation and western blot of MAP3K15–Dorsal interaction in shrimp cytoplasm at 24 h post-WSSV, following SDK1 or PBS pretreatment. (B-D) Western blot analysis of nuclear STAT and Dorsal levels (B), phosphorylated STAT (C), and CC-CL expression (D) in the intestine of L. vannamei following SDK1 treatment and WSSV infection. (E) The expression levels of wsv249, wsv100, wsv403, wsv107, and wsv069 in the intestine of L. vannamei were analyzed by qPCR at 6 hours post-WSSV infection following SDK1 treatment. Data are presented as the mean ± SD from three independent replicates and were analyzed by Student’s t-test. *, P < 0.05. **, P < 0.01. β-actin was used as the internal reference. Western blot bands were quantified using ImageJ, with values representing relative band intensities. (TIF) [file ppat.1013349.s008.tif]
