# Supplementary material for: MAP3K15 facilitates multiple viral genes expression in crustaceans via Dorsal-CC-CL-STAT axis besides the JNK/P38 pathway
Source: PLoS Pathog. 2025 Aug 1;21(8):e1013349. doi: 10.1371/journal.ppat.1013349 (PMC12316391; doi:10.1371/journal.ppat.1013349)
Supplement: S1 Table — (DOCX) [file ppat.1013349.s009.docx]

**S1 Table.** **Sequence of primers used in this study.**

| **Primers** | **Sequence (5' - 3')** |
| --- | --- |
| **Recombinant expression** | |
| MAP3K15-exF | GCCATGGCTGATATCGGATCCAGCAGCATCAAGTCCATAAC |
| MAP3K15-exR | GTGGTGGTGGTGGTGCTCGAGGAGAGTTGTCCGTCGCTGA |
| CC-CL-exF | GCCATGGCTGATATCGGATCCATGTGGCCGTGTGTATTGATTC |
| CC-CL-exR | GTGGTGGTGGTGGTGCTCGAGAAACAGATGACAGATATAGGGC |
| **qPCR** |  |
| MAP3K15-RTF | TGGAAAGCGCCTTGTACTGG |
| MAP3K15-RTR | AAGAAGCCATCCTCTGACAC |
| ie1-RTF | GCCAGTTGTATCAGCGAA |
| ie1-RTR | TATGCTCCGCCCTCCTTC |
| vp28-RTF | AGCTCCAACACCTCCTCCTTCA |
| vp28-RTR | TTACTCGGTCTCAGTGCCAGA |
| CC-CL-RTF | TGAGGCGAAACTAAACAAACATG |
| CC-CL-RTR | GTGAGGCGGTCTTCCAAACG |
| Prx1-RTF | GAGGATGAGGGCATTGCTT |
| Prx1-RTR | CAGTGGGATCAGCCTTCATT |
| Prx4-RTF | CTATGGGGTCTACCTGGAG |
| Prx4-RTR | TGCTGGTCTGTGAACTGG |
| Dorsal-RTF | GCAATGCTGGTAACCTGGCTA |
| Dorsal-RTR | CTATGGGATTTTGGTCAATACACTTT |
| STAT-RTF | GGTCCCAGTTCTGTAAGG |
| STAT-RTR | TAGGCACATTCGGATAAA |
| *wsv249*-RTF | GGCACATTGGGAGGTAATTT |
| *wsv249*-RTR | TTTCCGTACACATCAGCAGA |
| *wsv100*-RTF | AGATTCTTGGCTGAACATGG |
| *wsv100*-RTR | CAAAGCATACGACAACTCCA |
| *wsv403*-RTF | GACCAGTTCCAACCCAAGAA |
| *wsv403*-RTR | GCAGTGGAATAGCAAGGGAA |
| *wsv107*- RTF | ACTCAAATCGTCTGGAACCT |
| *wsv107*-RTR | ATCCTCAATAAACGGGCTTC |
| *wsv069*-RTF | AATATGGACTTGACGGCTGG |
| *wsv069*-RTR | ATACGACATAGCACCTCCAC |
| JNK-RTF | ATGGGCTACACAGAGAACGT |
| JNK-RTR | GGTCGAAGGAATAGCCAGGG |
| P38-RTF | GCCCCACATGAGGAAGAAGG |
| P38-RTR | GGCTCACTGTCAGGCTCATC |
| *β*-actin-RTF | CAGCCTTCCTTCCTGGGTATGG |
| *β*-actin-RTR | GAGGGAGCGAGGGCAGTGATT |
| **RNAi** |  |
| MAP3K15RNAiF | GCGTAATACGACTCACTATAGGAGATCTTCATGGAGCAGGT |
| MAP3K15RNAiR | GCGTAATACGACTCACTATAGGTCAAGCACACTCCAGCCA |
| Dorsal-RNAiF | GCGTAATACGACTCACTATAGGCCATAGAGCTAGATA |
| Dorsal-RNAiR | GCGTAATACGACTCACTATAGGTCAGTACCCAAGTGT |
| STAT-RNAiF | GCGTAATACGACTCACTATAGGACTTTCCTGCTCCGT |
| STAT-RNAiR | GCGTAATACGACTCACTATAGGCGTTGGCACTGTTGAGAC |
| CC-CL-RNAiF | GCGTAATACGACTCACTATAGGCAACCCGCCCGGCACCC |
| CC-CL-RNAiR | GCGTAATACGACTCACTATAGGAGAATAAATTAATATGGC |
| *wsv249*-RNAiF | GCGTAATACGACTCACTATAGGGGATTCAGAGACCATGGCTT |
| *wsv249*-RNAiR | GCGTAATACGACTCACTATAGGCGGCCTTTTCTTCAAATTCT |
| *wsv069*-RNAiF | GCGTAATACGACTCACTATAGGAGTGTGGGAATTTTGAAGCA |
| *wsv069*-RNAiR | GCGTAATACGACTCACTATAGGAAATATCCCCCACGTGAACC |
| *wsv100*-RNAiF | GCGTAATACGACTCACTATAGGTGGAGTTGTCGTATGCTTTG |
| *wsv100*-RNAiR | GCGTAATACGACTCACTATAGGGGAAGTGGGTTGGTATTCAC |
| *wsv107*-RNAiF | GCGTAATACGACTCACTATAGGTTTCTGACCACTTGTGCATC |
| *Wsv107*-RNAiR | GCGTAATACGACTCACTATAGGCCCTTAATACGTTTCCAATCCT |
| JNK-RNAiF | GCGTAATACGACTCACTATAGGCAAAGCAAGTCAAGCACGAG |
| JNK-RNAiR | GCGTAATACGACTCACTATAGGGATGAGCTCCTTCCACTGGT |
| P38-RNAiF | GCGTAATACGACTCACTATAGGGTGAGCTGAAGATCCTCGAC |
| P38-RNAiR | GCGTAATACGACTCACTATAGGCGTCCCAACCAGCTTCATAA |
| GFP-RNAiF | GCGTAATACGACTCACTATAGGTGGTCCCAATTCTCGTGGAAC |
| GFP-RNAiR | GCGTAATACGACTCACTATAGGCTTGAAGTTGACCTTGATGCC |
| **ChIP** |  |
| *wsv249*DorsalChIP-F | AAAAAGCTTTGGTTGGGG |
| *wsv249*DorsalChIP-R | ATCCTCTGTATGGTCCGC |
| *wsv100*DorsalChIP-F | GTCCTAAGAAAGATGAGC |
| *wsv100*DorsalChIP-R | GTATCATTTGAGTCGGTC |
| *wsv051*DorsalChIP-F | AGTTGAGGTTAAGTTTGAAATTG |
| *wsv051*DorsalChIP-R | TTTATTATTTTTGCCGTCTC |
| *wsv303*DorsalChIP-F | TTCTCCCATTCCTTCAGC |
| *wsv303*DorsalChIP-R | CTACTGAATGAACAGATGCG |
| *wsv079*DorsalChIP-F | GGGCGTTGAATTAATCGT |
| *wsv079*DorsalChIP-R | ACTGCTGGTGGTGGTGGT |
| *wsv069*DorsalChIP-F | GAGGGGTATCAACCACAC |
| *wsv069*DorsalChIP-R | ACAACAGCGATTACACCA |
| *wsv*2*49*STATChIP-F | CCTTAGGCGAGTCATGTTTCT |
| *wsv249*STATChIP-R | TATACACCCAGATCGGCACA |
| *wsv100*STATChIP-F | AACAGAACCAACAATGCTCA |
| *wsv100*STATChIP-R | ATCACGCTTCTTGTTATAGGG |
| *wsv051*STATChIP-F | CGCGCCAGAGCATAGATCTT |
| *wsv051*STATChIP-R | CGCCCCCAATTTTTATTCCG |
| *wsv403*STATChIP-F | AGCCACATCTGCGTCATACA |
| *wsv403*STATChIP-R | AAAACAAACCTCCCACCCTC |
| *wsv303*STATChIP-F | GGGGTTTCTTTGGCTTCC |
| *wsv303*STATChIP-R | TCACTAGCGAGAGAGACA |
| *wsv079*STATChIP-F | TTAGATGGTGGGGAGTAT |
| *wsv079*STATChIP-R | AGTTCTTCCGTGTTCCTA |
| *wsv107*STATChIP-F | AGACCCCTGACCTAAAGCAG |
| *wsv107*STATChIP-R | GATATCACCAAGTCACCCCTT |
| *wsv083*STATChIP-F | ATCTTCTCGTGCTCCTCG |
| *wsv083*STATChIP-R | GCCAGCAGAAGAGCAACA |
| *wsv069*STATChIP-F | AGGTGTTAAAGAAGCAGTTGT |
| *wsv069*STATChIP-R | ACACAAATATGCTCCGCCCA |
| CC-CLDorsalChIP-F | CAAGATCTAACATATCCGGTTG |
| CC-CLDorsalChIP-R | GCGGAAGAAAATAAACAGGGT |
